# Extreme deviations from the normative model reveal cortical heterogeneity and associations with negative symptom severity in first-episode psychosis from the OPTiMiSE and GAP studies

**DOI:** 10.1038/s41398-023-02661-6

**Published:** 2023-12-02

**Authors:** Amanda Worker, Pierre Berthert, Andrew J. Lawrence, Seyed Mostafa Kia, Celso Arango, Richard Dinga, Silvana Galderisi, Birte Glenthøj, René S. Kahn, Anoushka Leslie, Robin M. Murray, Carmine M. Pariante, Christos Pantelis, Mark Weiser, Inge Winter-van Rossum, Philip McGuire, Paola Dazzan, Andre F. Marquand

**Affiliations:** 1 Department of Psychological Medicine, Institute of Psychiatry, Psychology and Neuroscience, King’s College London, London, UK; 2 Department of Psychology, University of Oslo, Oslo, Norway; 3 Norwegian Center for Mental Disorders Research (NORMENT), University of Oslo, and Oslo University Hospital, Oslo, Norway; 4 Donders Institute for Brain, Cognition, and Behavior, Radboud University, Nijmegen, the Netherlands; 5 Department of Cognitive Neuroscience, Radboud University Medical Center, Nijmegen, the Netherlands; 6 Department of Cognitive Science and Artificial Intelligence, Tilburg University, Tilburg, the Netherlands; 7 Department of Child and Adolescent Psychiatry, Institute of Psychiatry and Mental Health, Hospital General Universitario Gregorio Marañon, IiSGM, CIBERSAM, School of Medicine, Universidad Complutense Madrid, Madrid, Spain; 8 University of Campania “Luigi Vanvitelli”, Naples, Italy; 9 Center for Clinical Intervention and Neuropsychiatric Schizophrenia Research (CINS) and Center for Neuropsychiatric Schizophrenia Research (CNSR), Mental Health Center, Glostrup, Copenhagen University Hospital – Mental Health Services CPH, Copenhagen, Denmark; 10 Department of Clinical Medicine, Faculty of Health and Medical Sciences, University of Copenhagen, Copenhagen, Denmark; 11 Department of Psychiatry, Icahn School of Medicine at Mount Sinai, New York, NY USA; 12 Department of Neuroimaging, Institute of Psychiatry, Psychology and Neuroscience, King’s College London, London, UK; 13 Department of Psychosis Studies, Institute of Psychiatry, Psychology and Neuroscience, King’s College London, London, UK; 14 National Institute for Health Research Mental Health Biomedical Research Centre, South London and Maudsley National Health Service Foundation Trust and King’s College London, London, UK; 15 Biological Psychiatry, Institute of Psychiatry, Psychology and Neuroscience, King’s College London, London, UK; 16 Melbourne Neuropsychiatry Centre, Department of Psychiatry, The University of Melbourne and Melbourne Health, Carlton South, Victoria, Australia; 17 Department of Psychiatry, Sheba Medical Center, Tel Hashomer, Tel Aviv, 52621 Israel; 18 Sackler School of Medicine, Tel Aviv University, Tel Aviv, Israel; 19 Department of Psychiatry, University Medical Center Utrecht Brain Center, Utrecht University, Utrecht, The Netherlands

**Keywords:** Molecular neuroscience, Prognostic markers, Schizophrenia, Bipolar disorder

## Abstract

There is currently no quantifiable method to predict long-term clinical outcomes in patients presenting with a first episode of psychosis. A major barrier to developing useful markers for this is biological heterogeneity, where many different pathological mechanisms may underly the same set of symptoms in different individuals. Normative modelling has been used to quantify this heterogeneity in established psychotic disorders by identifying regions of the cortex which are thinner than expected based on a normative healthy population range. These brain atypicalities are measured at the individual level and therefore potentially useful in a clinical setting. However, it is still unclear whether alterations in individual brain structure can be detected at the time of the first psychotic episode, and whether they are associated with subsequent clinical outcomes. We applied normative modelling of cortical thickness to a sample of first-episode psychosis patients, with the aim of quantifying heterogeneity and to use any pattern of cortical atypicality to predict symptoms and response to antipsychotic medication at timepoints from baseline up to 95 weeks (median follow-ups = 4). T1-weighted brain magnetic resonance images from the GAP and OPTiMiSE samples were processed with Freesurfer V6.0.0 yielding 148 cortical thickness features. An existing normative model of cortical thickness (*n* = 37,126) was adapted to integrate data from each clinical site and account for effects of gender and site. Our test sample consisted of control participants (*n* = 149, mean age = 26, SD = 6.7) and patient data (*n* = 295, mean age = 26, SD = 6.7), this sample was used for estimating deviations from the normative model and subsequent statistical analysis. For each individual, the 148 cortical thickness features were mapped to centiles of the normative distribution and converted to z-scores reflecting the distance from the population mean. Individual cortical thickness metrics of +/– 2.6 standard deviations from the mean were considered extreme deviations from the norm. We found that no more than 6.4% of psychosis patients had extreme deviations in a single brain region (regional overlap) demonstrating a high degree of heterogeneity. Mann-Whitney U tests were run on z-scores for each region and significantly lower z-scores were observed in FEP patients in the frontal, temporal, parietal and occipital lobes. Finally, linear mixed-effects modelling showed that negative deviations in cortical thickness in parietal and temporal regions at baseline are related to more severe negative symptoms over the medium-term. This study shows that even at the early stage of symptom onset normative modelling provides a framework to identify individualised cortical markers which can be used for early personalised intervention and stratification.

## Introduction

There is a pressing need to develop quantifiable marker(s) to enable early diagnosis and optimised individual care programmes for people with psychotic disorders [1]. The course of illness is highly heterogeneous after a first episode of psychosis (FEP). The diagnosis of psychosis is currently entirely based on symptoms, which are highly variable between individuals and overlap substantially across diagnoses of schizophrenia and affective disorders. While many people make a full recovery after the first episode of psychotic illness, others experience ongoing symptoms, and a later diagnosis of schizophrenia, bipolar disorder, and other related disorders. Furthermore, while antipsychotic medications remain the first line of treatment in FEP, up to 30% of patients do not respond to these drugs [2]. To date, there is no reliable way for clinicians to predict the course of the disorder, after the first contact with psychiatry services.

Neuroimaging is a promising tool for identifying neurobiological markers of illness in psychotic disorders. The current ideal is that neuroimaging-derived markers could enable early identification of illness course, thus leading to personalised interventions at an early stage. However, neuroimaging has so far largely failed to produce the results expected. This is due to a lack of visible pathology on magnetic resonance imaging (MRI), small sample sizes and methods commonly adopted (e.g. region of interest analysis). Nonetheless, several studies have found differences in brain structure in FEP. For example, Vieira and colleagues found widespread smaller volumes in FEP compared to controls, notably in the gyrus rectus region, which was also negatively correlated with positive and negative psychotic symptoms [3]. Damjaha and colleagues also found that an enlarged orbitofrontal cortex and superior temporal gyrus are associated with primary negative symptoms, while another study identified an association between the superior temporal, right parahippocampal and left orbitofrontal gyri and persistent negative symptoms [4, 5]. This kind of cross-sectional analysis can be useful for identifying potential markers but for it to be clinically useful we must establish whether brain markers can predict clinical outcomes over the medium and long term. Using longitudinal clinical follow-ups Dazzan and colleagues found that regional cortical thickness was not associated with remission symptoms over the medium term, but the more complex metric of structural connectivity showed some association with remission at 74 weeks [6].

It is now understood that the presence of very subtle and heterogeneous neuro-morphological patterns in the brain exists in psychiatric disorders. It is widely accepted that this biological heterogeneity is a leading cause of the lack of progress made in elucidating the mechanisms underlying the pathophysiology and course of psychosis. Heterogeneous patterns are not detected using the standard case-control approach adopted by most neuroimaging studies [7], which largely focuses on group differences. While case-control analysis enables us to conclude the average patient, it does not allow inferences to be made for individuals, which is an essential component to building a clinically useful tool.

Normative modelling provides the opportunity to make these crucial individual-level inferences and identify individualised clinical risk profiles, which is a key focus for this work and a critical step towards true precision medicine in psychotic disorders. To this end, normative modelling has been used to quantify brain structural heterogeneity, using regional overlap, in adults with established schizophrenia and bipolar disorder (Wolfers et al., 2018, 2021). Regional overlap is a calculation of how many people within a group (e.g. patient group) display brain atypicality (i.e. deviation from the norm) in a single region or voxel, expressed as a percentage. Positive deviations (increases relative to the normative model) and negative deviations (decreases) are usually calculated separately for each region. For example, a low percentage of negative deviations in a single region denotes that few individuals have the same pattern of extreme negative deviations in that region, which represents a high degree of heterogeneity. Previous studies have found little overlap between patients in the brain loci associated with each disorder e.g. less than 2% of schizophrenia patients have extreme deviations in the same brain voxels across the majority of the grey matter, few brain loci exceed this overlap with up to 8% of patients displaying extreme deviations, the picture is similar for bipolar disorder [8]. This demonstrates the complexity of the underlying biological features and the need to model interindividual differences to quantify individual clinical risk. To date, brain structural heterogeneity has not been quantified in this way in first-episode psychosis.

This study sought to address this gap in the literature by applying normative modelling to investigate heterogeneity in cortical thickness across the whole cortical mantle. In this sample of adults with psychosis, we aimed to (1) quantify cortical thickness heterogeneity by mapping regional cortical thickness deviations at the individual level, (2) assess group-level differences in deviations from the expected pattern of cortical thickness between patients and control participants, and (3) evaluate the relationship between cortical thickness deviations and symptom severity (PANSS), treatment response and clinical outcome both at baseline and over the medium term (0–95 weeks, median timepoints = 4).

A key component in quantifying meaningful deviations is to use a representative reference normative range. To achieve this, large multi-site datasets are necessary, which is challenging due to well-documented site effects in brain MRI data [9]. In the present study, we used one of the largest (*n* = ~40,000) reference normative models available, built using a federated, hierarchical Bayesian regression approach across a population of multi-site MRI data with an age range of 6–100 years [10]. This federated learning approach enabled us to adapt the parameters of the reference normative model to our first-episode psychosis samples in situ, i.e. without the need to transfer sensitive clinical data. Using this method, site and gender effects are removed and the resulting z-scores from multiple sites are comparable and can be combined into a single, larger sample for statistical analysis (i.e. removing the need to include these variables in later models and enabling simpler univariate models to be employed). Furthermore, this approach avoids a common pitfall of “controlling” for variables such as site. This process involves the necessary removal of variation associated with the site but likely results in the unintentional removal of variance that correlates with a site such as age (e.g. if a single site collects data from a specific age range, the site effect correlates with age), and unknown but important biological variance such as subtypes within a diagnostic category (see Kia et al., 2020, 2022 for further discussion). In summary, this approach has several strengths which enable multi-site analysis of cortical thickness concerning a normative range and simplify later statistical analysis (e.g. linear mixed effects modelling). Note that since none of the sites in the GAP and OPTIMISE studies were included in the reference model, it is necessary to perform the model adaptation step where held-out control data are used to learn the site effect while avoiding inadvertently removing clinically relevant effects.

We hypothesised that (1) highly individualised patterns of deviations would be observed in individuals with psychosis, thus demonstrating the potential for normative modelling to identify neurobiological atypicality even at the early stages of symptomatic illness, and (2) these patterns of cortical atypicality would be predictive of short to medium term symptom severity following treatment with antipsychotic medications.

## Methods

### Participants

We included data from two large samples of individuals who were recruited and assessed at the time of their first presentation to services in the Genetics and Psychosis (GAP) study, and the Optimization of Treatment and Management of Schizophrenia in Europe (OPTiMiSE) study.

#### GAP

A total of 128 patients were recruited when presenting for the first time with an episode of psychosis to services in the South London and Maudsley National Health Service Foundation Trust, Southeast London, England. FEP patients eligible for the study were presenting with symptoms in the following areas that would score 4 or higher on the Positive and Negative Syndrome Scale (PANSS): delusions, hallucinations, thought disorder, or negative symptoms of schizophrenia. These symptoms must have lasted seven days or longer. Patients were diagnosed by a psychiatrist using ICD-10 criteria and this sample includes patients with: bipolar disorder, major depression with psychotic symptoms, schizophrenia, schizophreniform disorder, schizoaffective disorder and psychosis not otherwise specified. At the time of recruitment to the study, patients were in the early phase of symptomatic illness and therefore minimally treated with antipsychotic medication. A control sample of 94 individuals with no history of psychosis was recruited from the same geographical area and matched for age, sex, ethnicity, educational qualifications, and employment status. All subjects provided written informed consent and the study was approved by the ethical review boards of sites contributing data. Further details can be found in previous publications [11, 12].

As there were fewer control participants in the older age range in the GAP sample, additional data (*n* = 124) from control participants in the ATLAS study were used to supplement the sample. The ATLAS study was designed to map brain changes and cognition across the healthy adult lifespan.

OPTIMISE (multi-site)

A total of 192 FEP patients were recruited to the neuroimaging component of the clinical trial at participating centres after referral from nearby healthcare facilities and through public advertisements. Patients were eligible for the study if they were aged 18–40 years and met DSM-IV criteria for schizophrenia, schizophreniform disorder, or schizoaffective disorder, which was confirmed by the Mini International Neuropsychiatric Interview. At the time of recruitment to the study, patients were in the early phase of symptomatic illness and therefore minimally treated with antipsychotic medication. A reference sample of control participants was recruited from the same geographical area for each site that participated in the neuroimaging component of the clinical trial (*n* = 114). All patients provided written informed consent and the study was approved by the local ethical review boards at each site contributing data. Further details can be found in previous publications [2, 13].

### Clinical data

At baseline, patients were assessed using the Positive and Negative Scale for Schizophrenia (PANSS). PANSS assessments were also conducted at various time points throughout the study; GAP assessments were done at 12 weeks and 1 year while assessments for OPTIMISE were conducted at various time points up to 95 weeks (4 (median) follow-up assessments). PANSS scores were calculated in the following domains: positive, negative and general and summary scores, which were computed as the sum of items within each domain. Remission status was defined as a reduction in symptom severity to the levels set out by the Schizophrenia Working Group Consensus [14]. Remission was achieved at each time point if the PANSS rating was mild or less (score of 1, 2 or 3) in the following items: delusions (P1), unusual thought content (G9), hallucinatory behaviour (P3), conceptual organization (P2), mannerisms/posturing (G5), blunted affect (N1), social withdrawal (N4), and lack of spontaneity (N6). As these items are deemed to be the most important for measuring remission status but using the binary variable of remission status reduces the available statistical power, these items were also summed to create a remission score with increased statistical power compared to the binary remission status. This was an exploratory approach used in addition to remission status (in separate models), and we recognise that changes above/below the defined threshold for one or more items would cause divergence from remission status, thus the two variables are not directly interchangeable.

### Image acquisition and processing

#### GAP

Participants underwent MRI scanning as soon as possible after first contact with psychiatric services to limit exposure to antipsychotic medication at the time of the scan. All MRI scans were acquired in a 3-T Signa HDx scanner (General Electric) at the Centre for Neuroimaging Sciences, Institute of Psychiatry, Psychology and Neuroscience, King’s College London. A sagittal 3-dimensional magnetization prepared rapid-acquisition gradient-echo volumetric scan was obtained from each subject. The scan had an image matrix size of 256 × 256 × 166 voxels, with an in-plane voxel size of 1.02 × 1.02 mm and a slice thickness of 1.2 mm (echo time, 2.848 milliseconds; repetition time, 6.988 milliseconds; inversion time, 650 milliseconds; excitation flip angle, 20°; 1 data average). The ATLAS data were collected within the same timeframe, on the same scanner and shared the above acquisition parameters and are reported as part of the GAP sample from herein.

#### OPTiMiSE

All sites acquired 3T sagittal T1-weighted structural MRI with whole-brain coverage at 1 × 1 × 1.2 mm resolution using parameters optimised for multi-site data acquisition and made available by the Alzheimer’s Disease Neuroimaging Initiative (http://adni.loni.usc.edu/). Additional technical details for MR scanners and sequences are provided in Supplementary materials (Table S1 & Table S2). MR images were transferred to a coordinating centre (King’s College London, London, UK) for processing and analysis. Images were converted from their scanner format (DICOM, or PAR/REC) into axially oriented NIFTI using MRIcroGL dcm2nii (https://www.mccauslandcenter.sc.edu/mricrogl) and cropped in the axial dimension to exclude the neck.

**Fig. 1:**
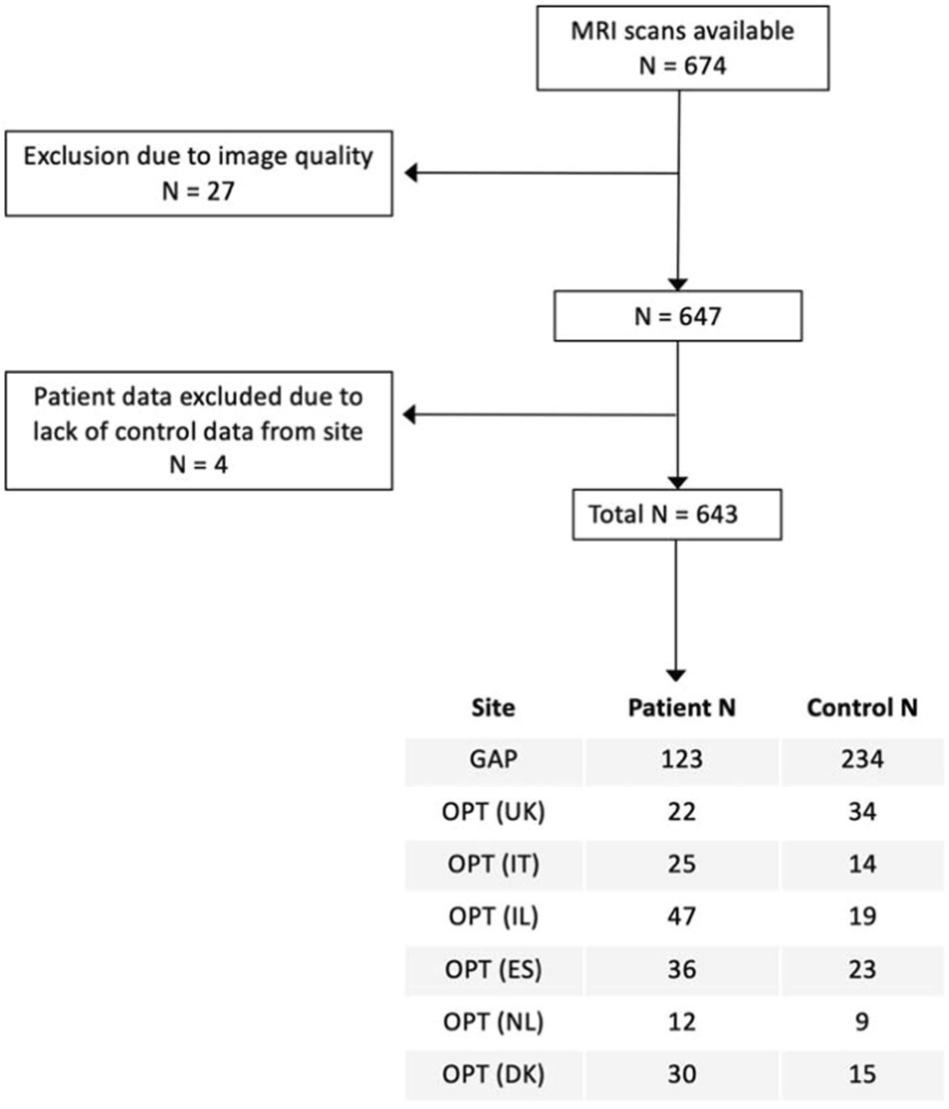
Participant inclusion workflow. Flowchart showing final sample selection.

Both GAP and OPTiMiSE T1-weighted images were visually inspected by an experienced neuroimaging researcher and 21 images were excluded due to poor image quality. A further 4 patient images were excluded due to a lack of control data for that site, which was necessary for the adaptation of the normative model (see Fig. 1). All T1-weighted images were processed with Freesurfer version 6.0 (http://surfer.nmr.mgh.harvard.edu/) for cortical reconstruction, parcellation and estimation of regional morphometric measures. For each of the 148 regions of the Destrieux atlas, cortical thickness was calculated and extracted.

### Normative modelling and statistical analysis

We used a pre-estimated multi-site reference normative model predicting cortical thickness from age. This model was built from healthy participant data (*n* = 37,126) across 79 scanners with good coverage through adolescence to late life [15] in order to maximise the coverage over the lifespan. Participant sex and scanner site have been accounted for in the model by inclusion as batch effects which can be interpreted as levels in multi-level modelling, enabling nuisance variance related to site and sex to be removed. Note that we have shown previously that a Gaussian model provides a reasonable fit to cortical thickness estimates [15]. In order to apply this model to our clinical samples, it is necessary to perform a model adaptation step to optimise the fit to the unseen scanners, i.e. to learn and account for scanner effects. This has been described in detail in prior publications [15–17]. Briefly, the parameters of this reference normative model were adapted to the new sites with 50% of control participant data from each clinical site from the OPTiMiSE sample, in line with recommendations from prior work [18]. The adaptation samples were then discarded in order to prevent overfitting. The GAP sample (prior to inclusion of ATLAS data) includes a small number of patients over the age of 45 (*n* = 3), therefore random selection of 50% controls resulted in non-random random selection with a bias towards the more densely populated, younger age range. To ensure that the older age range from the GAP site was appropriately represented in the normative model the ATLAS data was selected as the adaptation set, which is slightly over 50% of available control data. For both samples, the remaining control participant and patient data were used as test samples for estimating deviations from the normative range. These deviations are quantified as individual level, region specific z-scores. A threshold of +/– 2.6 (*p* < 0.01) was used to identify extreme deviations.

To address aim 1, regional overlap (representing the percentage of patients or controls displaying extreme deviations) was calculated and overlayed for each region, for example computing (N extreme negative deviations in patients/N patients * 100) to yield regional overlap of extreme deviations for patients. The proportion of extreme negative/positive deviations for patients/controls was calculated (N extreme negative deviations in patients/(N patients * N regions) * 100) for example. For aim 2, we tested whether there were differences in regional z-scores between patients and control participants using a Mann-Whitney U test for each region [19] and controlling for multiple comparisons using the Benjamini-Hochberg method [20].

To test for a relationship between baseline brain atypicalities and longitudinal PANSS scores and remission status [14] a longitudinal generalised linear mixed effects (LME) model was built using the lmer and glmer functions in the lmerTest and lmer4 packages implemented in R version 4.1.1. Separate models were run for each region, with time from baseline, measured in weeks, and regional z-score as fixed effect, in addition to random intercept and slope terms for subject and time. Dependent variables (in separate Gaussian models) were the sum of negative PANSS items, positive PANSS items, general PANSS items and items used to calculate remission status according to Andreason criteria (lmer with lmerTest). A separate binomial linear mixed effects model was built with remission status as the dependent variable (glmer and lmer4). Where necessary, the problem of multiple comparisons across regions was controlled for using Benjamini-Hochberg method [16].

Additional checks were performed by analysing the GAP and OPTiMiSE datasets separately, by excluding sites with fewer than 10 control participants in the test data.

## Results

A total of 295 patients with FEP (GAP *N* = 123, OPTiMiSE *N* = 172) were included (mean [SD] age, 26 [6.7] years; 143 [49%] male). 348 control participants were also included in the study. 199 control participant brain scans were used for adaptation of the normative reference model (mean [SD] age, 35 [16.3] years; 87 [44%] male), resulting in a test sample consisting of the 295 FEP patients and 149 control participants (mean [SD] age, 26 [6.7] years; 60 [40%] male) (see Table 1 and Table S3 for site breakdown).

**Table 1:** Demographics and clinical data.

	All patients (295)	All healthy participants (149)
Age, mean years (s.d.)	26.13 (6.72)	26.50 (6.72)
Sex, N male, (%)	143 (49%)	60 (40%)
Ethnicity		
N white (%)	192 (65.1)	82 (55.0)
N black (%)	56 (19.0)	32 (21.5)
N Asian (%)	18 (6.1)	1 (0.6)
N other (%)	28 (9.5)	13 (8.7)
N unknown (%)	1 (0.3)	21 (14.1)
Clinical follow-up		
Assessments taken at weeks median, (min, max)	4 (0, 95)	–
Number of longitudinal assessments, median, (min, max)	4 (1, 15)	–
PANSS baseline, N	266	–
Positive median, (min, max)	17 (7, 35)	–
Negative median, (min, max)	15 (7, 36)	–
General median, (min, max)	33 (7, 36)	–

### Spatial extent of extreme deviations from the expected pattern (normative model)

**Fig. 2:**
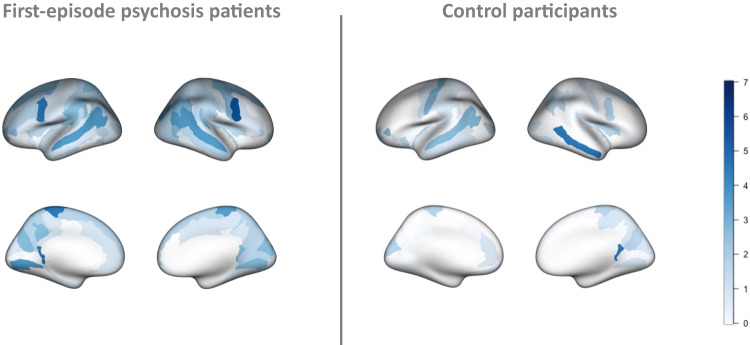
Regional overlap of extreme negative deviations from the normative models in first episode psychosis patients relative to controls. Percentage overlap, in first-episode psychosis patients and control participants, of extreme negative deviations from the normative model for each brain region (Destrieux atlas) (*p* < 0.01).

**Fig. 3:**
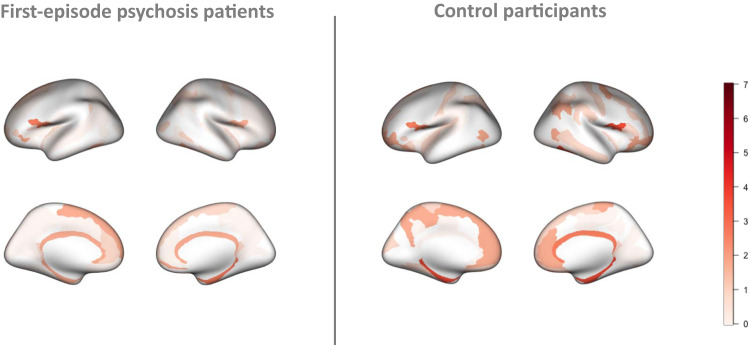
Regional overlap of extreme positive deviations from the normative model in first episode psychosis patients relative to controls. Percentage overlap, in first-episode psychosis patients and control participants, of extreme positive deviations from the normative model for each brain region (Destrieux atlas) (*p* < 0.01).

Regional overlap of extreme negative deviations did not exceed 6.4% in any brain region in patients and 5.4% in control participants, providing evidence of inter-individual heterogeneity across individuals. The regions with overlap greater than 5% in patients were: bilateral inferior precentral gyrus, left post-ventral cingulate and left paracentral gyrus (Fig. 2). Overlap of extreme positive deviations did not exceed 3.7% in patients and 4.7% in controls (Fig. 3).

### Group comparisons

Patients with FEP displayed a higher percentage of extreme negative deviations across the 148 atlas regions (1.77%) than control participants (0.77%) (Fig. 2). Conversely, control participants displayed a higher percentage of extreme positive deviations across the cortical regions (0.88%) than patients (0.59%) (Fig. 3).

**Fig. 4:**
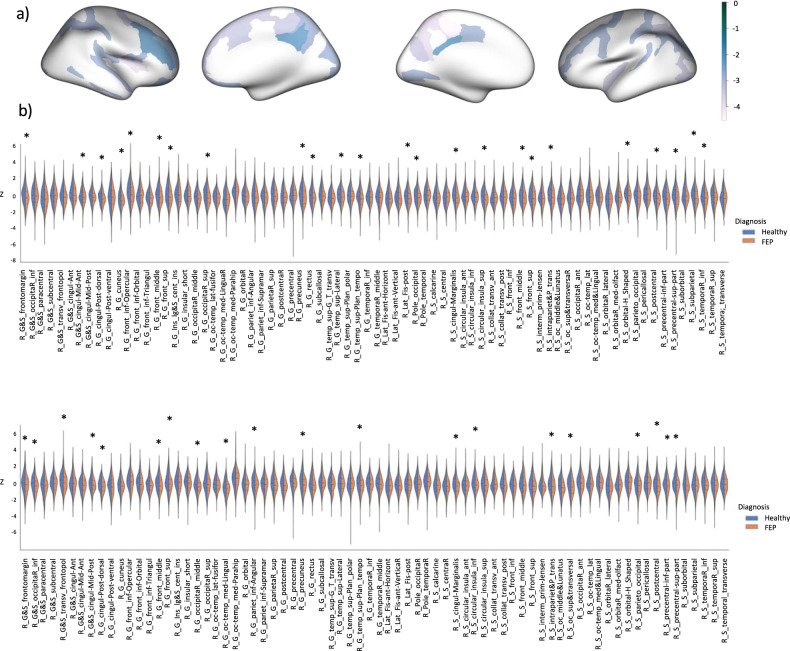
Regional distribution of differences in median deviations in patients relative to controls. Results from regional Mann-Witney U tests comparing median z-score between FEP patients and healthy participants. **a** regional standardised effect sizes overlaid on glass brain, **b** violin plots displaying regional z-score distributions and significant regions after Benjamin-Hochberg correction for multiple comparisons (*p* < 0.05).

A Mann-Whitney U test was run on the z-scores of each brain region to determine if there were differences in median z-score between FEP patients and control participants. Distributions of the Z-scores were similar, as assessed by visual inspection. Z-scores were significantly lower in FEP patients in regions across the frontal, temporal, parietal and occipital lobes, using an exact sampling distribution for U, after Benjamini-Hochberg correction for multiple comparisons (*p* < 0.05) (see Fig. 4). Note that the regions detected in the group analysis have a different spatial distribution than the individual deviations, providing further evidence of inter-individual heterogeneity.

### Prediction of clinical outcome from cortical thickness deviations

#### Symptom severity

**Fig. 5:**
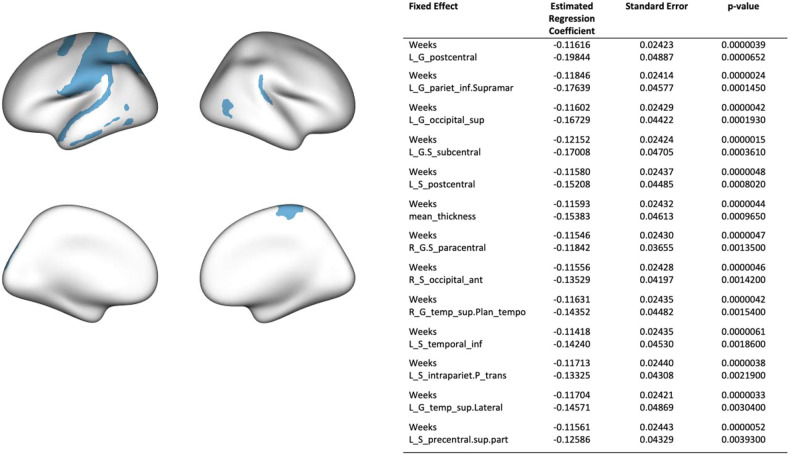
Prediction of symptoms at medium term follow-up from deviations from the normative models at baseline. Results from the linear mixed effects model predicting medium-term negative symptoms from weeks from baseline and regional z-scores. Significant regions are overlaid on a glass brain (FDR corrected).

There was a significant effect of both time (weeks) and mean cortical thickness z-score on negative PANSS scores, with negative symptoms improving over time (*p* = 4 × 10^–6^, coefficient = –0.116) and a thinner cortex relative to the reference range at baseline predicting more severe symptoms (*p* = 0.001, coefficient = –0.154). The LME model for each individual region showed that the following regions remained significant after correction for multiple comparisons: left postcentral gyrus (*p*-value < 0.001, coefficient = –0.198) and sulcus (*p*-value < 0.001, coefficient = –0.152), left inferior parietal gyrus (*p*-value < 0.001, coefficient = –0.176), left superior occipital gyrus (*p*-value < 0.001, coefficient = –0.167), left subcentral gyrus (*p*-value < 0.001, coefficient = –0.170), right paracentral gyrus (*p*-value < 0.01, coefficient = –0.118), right anterior occipital sulcus (*p*-value < 0.01, coefficient = –0.135), right superior temporal gyrus (*p*-value < 0.01, coefficient = –0.144), left inferior temporal sulcus (*p*-value < 0.01, coefficient = –0.142), left intraparietal sulcus (*p*-value < 0.01, coefficient = –0.133), left superior temporal gyrus (*p*-value < 0.01, coefficient = –0.146) and left precentral sulcus (*p*-value < 0.01, coefficient = –0.126) (see Fig. 5 and Supplementary Table 4).

There was a significant effect of time (weeks) (*p* < 1.0 × 10^–5^, coefficient = –0.271) but no significant effect of mean cortical thickness z-score (*p* = 0.548, coefficient = –0.026) on positive PANSS score. Regional cortical deviations did not significantly predict positive PANSS scores (see Supplementary Table 5).

There was a significant effect of time (weeks) (*p* < 1.0 × 10^–5^, coefficient = –0.248) but no significant effect of mean cortical thickness z-score (*p* = 0.275, coefficient = –0.046) on general PANSS score. Regional cortical deviations did not significantly predict general PANSS scores (see Supplementary Table 6).

#### Remission status

There was a significant effect of time (weeks) and left central gyral deviations on remission status, with remission more likely over time (*p* = 0.0082, coefficient=0.433) and with patients with thinner cortex relative to the reference range being less likely to remit (*p* = 0.0004, coefficient=0.415, odds ratio=1.423) (see Supplementary Table 7). Twenty-three regions also demonstrated a nominally significant relationship with remission status, but these findings did not remain significant after correction for multiple comparisons.

There was a significant effect of time (weeks) and regional cortical thickness on the summed items used in the definition of remission, with time being predictive of improving symptoms and a thinner cortex relative to the reference range being predictive of more severe symptoms in seventeen regions (see Supplementary Table 8). However, these findings did not remain significant after correction for multiple comparisons using the Benjamini-Hochberg procedure.

### Sensitivity analyses

For these analyses we excluded sites with less than 10 control participants after adaptation. Patients with FEP displayed a significantly higher percentage of extreme negative deviations across the 148 atlas regions (2.29%) compared with control participants (0.87%, *p* < 0.0001, *X*^*2*^ = 129.17). Control participants displayed a higher percentage of extreme positive deviations across cortical regions (0.92%) than patients (0.71%, *p* = 0.014, *X*^*2*^ = 6.05). Regional overlap of extreme negative or positive deviations did not exceed 8% in any region. Median Z-scores were significantly lower in FEP patients than controls in sixty-two regions across the frontal, temporal, parietal and occipital lobes after Benjamini-Hochberg correction for multiple comparisons (see Supplementary Table 9).

## Discussion

In this study we assessed deviations in cortical thickness, with reference to one of the largest normative models available, in a sample of patients with first-episode psychosis (*n* = 295), quantifying neurobiological heterogeneity and estimating the association between cortical thickness deviations and symptom severity. Our main finding was that at the onset of psychosis, extensive deviations representing thinner than expected cortex are already evident across frontal, parietal, temporal, and occipital cortices. Moreover, the underlying neurobiological pattern is highly heterogeneous, with some extreme negative deviations detected in almost all brain regions and no more than 6.4% of patients displaying extreme deviations in the same region. We also found that FEP patients displayed a greater overall number of extreme negative deviations from the normative range than controls, which indicates widespread atypicality in patients. Finally, we found that negative deviations in cortical thickness in parietal and temporal regions at baseline were associated with more severe negative symptoms in the medium-term follow-up period (0-95 weeks).

To our knowledge, we are the first to report on cortical thickness, with reference to a large multi-site normative model, at both the group- and individual-level in FEP patients. Our findings support the notion of a high degree of biological heterogeneity in psychotic disorders. We demonstrate that at the time of psychosis onset, before patients have had long-term treatment, extreme cortical atypicalities are already present and highly variable between patients. These results are consistent with findings from previous studies where heterogeneity was demonstrated using a person-based similarity index in FEP [21] and low overlap in voxel-wise deviations using normative modelling in schizophrenia and bipolar disorder [8, 22]. These findings suggest that there are multiple pathophysiological pathways at play at the time of psychosis onset. The strength of our study over previous work is in our inclusion a large sample of patients in the very early stages of symptomatic illness and the large-scale normative model built from ~40,000 healthy subjects, which effectively manages site and gender effects while providing better representation of the true population range. Our results can be interpreted as deviations from the population norm and not simply the study norm.

We add to the existing body of literature on brain structure in FEP [3] and identify a pattern of overall atypicality in FEP. This atypical brain is reflected in two ways: 1) the number of widespread extreme deviations from the expected pattern of cortical thickness for the age and sex of our patient sample and 2) the difference in distribution of z-scores across forty-five regions, which captures not only extreme deviations but also more common subtle aberrations. Our results demonstrate the value of normative modelling in psychiatry research, particularly as the pattern of individualised extreme negative deviations highlights different cortical locations to the pattern observed at the group-level. This demonstrates that firstly, the underlying neurobiological mechanisms may be different across subsets of patients and secondly, that neural signatures (e.g. extreme deviations from the norm) which may be important clinical predictors are not captured by case-control analyses. While case-control analyses using the mean or median metric may be useful for identifying common patterns observed in most patients, normative modelling provides the opportunity to develop individualised clinical risk profiles which is a critical step towards precision medicine for psychotic and other mental disorders.

Twelve regions across the temporal and parietal lobes, where FEP patients had thinner than expected cortex, were identified as predictive of greater negative symptoms during the follow-up period. Negative symptoms in psychotic disorders are complex and are associated with particularly poor functional and clinical outcomes [23–27]. In this study we did not detect a significant relationship between medium-term positive symptoms and cortical atypicalities, which supports previous suggestions that negative and positive symptoms in FEP may have a different pathophysiological basis [28]. Studies have shown a pattern of heterogeneous and unstable positive symptoms over the long-term, which usually respond to antipsychotic treatment, while negative symptoms show a stable course which is linked to overall functioning and outcome [29, 30], indicating this stability may allow for more power in predictive analytics. The fact that no antipsychotic medication is unequivocally effective for negative symptoms has resulted in the emergence of clinical trials focusing on treatments targeting these symptoms [31]. However, at the first episode of psychosis, it is difficult to identify those who will suffer persistent negative symptoms and who may be most suitable for inclusion in these trials. Previous studies have identified regions associated with persistent and primary negative symptoms, including the superior temporal regions, consistent with our findings [4, 5]. Here, we identify a subset of patients with thinner cortex relative to the normative range who continue to have worse negative symptoms over time, despite antipsychotic medications. Thus, normative modelling of cortical thickness is a promising tool for identifying early neural markers of enduring negative symptoms.

The present study has notable strengths and advances our understanding in several ways. Firstly, the sample of FEP patients is relatively large in comparison to previous studies and patients were recruited immediately after first contact with psychiatry services and while antipsychotic use was still minimal. Thus, our findings demonstrate that biological differences exist at the early stage of symptomatic illness. Secondly, we use one of the largest normative models available. This model captures population-level age-related changes in cortical thickness across 148 brain regions and robustly integrates data from multiple sites yielding an accurate picture of the expected pattern of cortical thickness throughout adolescence and adulthood [10]. Assessing cortical thickness in FEP patients and control participants as centiles of variation from the expected pattern has permitted detection and mapping of distinct patterns of atypicality in individuals. Furthermore, we have demonstrated that normative modelling has clinical value by enabling the prediction of sustained negative symptoms over the medium term. We also found that it was possible to predict remission status from cortical thickness deviations, which was not possible using cortical thickness itself in the OPTiMiSE sample [6], thus demonstrating the increased power achieved by quantifying cortical thickness relative to the expected pattern for age and gender, in addition to longitudinal clinical follow-up. It has been shown previously that symptom remission at 12 weeks is an important predictor of symptom and functional recovery at 10 years [32] and therefore future studies may wish to assess whether cortical thickness deviations can predict longer-term outcomes.

A limitation of this study is that we are unable to confirm the degree to which confounding variables such as antipsychotic medications and ethnicity might have influenced the results. Some FEP patients were exposed to medications at the time of scanning, thus we cannot exclude any effects that medication might have had on cortical thickness. However, we can rule out the effects of chronic use of medication as all patients were minimally treated. Our large-scale normative model is built from datasets across Europe where ethnicity data is not always available or is measured in a crude way (e.g. white, black, Asian). We are therefore unable to make inferences at this time about the effects of ethnicity on our findings, but ongoing work aims to integrate ethnicity into the normative modelling framework in a similar way as it has been done for gender and site-effects [10]. Finally, negative symptoms are difficult to assess, and ideally this is done using specific scales for negative symptoms. In the present study, assessment of negative symptoms was done using the PANSS scale, thus further studies with more detailed analysis of negative symptoms will be necessary. Also, although highly significant, the effects that we report are of moderate magnitude and we consider that validation in prospective data is an important next step to assess the generalisability and clinical value of our findings.

The results from this study contribute to a body of research that aims to map the neurobiological heterogeneity of psychotic disorders, which is an important step towards achieving personalised medicine in psychiatry. The results from this study show that at the early stage of symptomatic illness, psychotic patients display patterns of widespread cortical atypicality. These atypicalities are highly heterogeneous between patients, demonstrating a complex and highly variable pattern of underlying pathophysiology. Moreover, the fact that patients with the greatest negative deviations in parietal and temporal regions are likely to have worse negative symptoms in the years following on from onset of symptoms, suggests that these individualised cortical markers could be used in the context of stratification for clinical trials on the efficacy of interventions targeting persistent, difficult to treat and life-altering negative symptoms.

### Supplementary information

Supplementary material

## Data Availability

Not all data are publicly shareable, but collaborative future projects are welcomed through reasonable request to the corresponding author.

## Code availability

All code for the estimation of the normative models is available online (https://github.com/amarquand/PCNtoolkit) and code for the subsequent statistical analysis is available from the corresponding author upon reasonable request.

## References

[1] Insel T, Cuthbert B, Garvey M, Heinssen R, Pine D, Quinn K (2010). Research Domain Criteria (RDoC): Toward a. Am J Psychiatry Online.

[2] Kahn RS, Winter van Rossum I, Leucht S, McGuire P, Lewis SW, Leboyer M (2018). Amisulpride and olanzapine followed by open-label treatment with clozapine in first-episode schizophrenia and schizophreniform disorder (OPTiMiSE): a three-phase switching study. Lancet Psychiatry.

[3] Vieira S, Gong Q, Scarpazza C, Lui S, Huang X, Crespo-Facorro B (2021). Neuroanatomical abnormalities in first-episode psychosis across independent samples: A multi-centre mega-analysis. Psychol Med.

[4] Demjaha A, Galderisi S, Glenthøj B, Arango C, Mucci A, Lawrence A, et al. Negative symptoms in First-Episode Schizophrenia related to morphometric alterations in orbitofrontal and superior temporal cortex: The OPTiMiSE study. Psychol Med. 2022. 10.1017/S0033291722000010 PMC10277764 35197142

[5] Bodnar M, Hovington CL, Buchy L, Malla AK, Joober R, Lepage M (2014). Cortical thinning in temporo-parietal junction (TPJ) in non-affective first-episode of psychosis patients with persistent negative symptoms. PLoS One.

[6] Dazzan P, Lawrence AJ, Reinders AATS, Egerton A, van Haren NEM, Merritt K (2020). Symptom remission and brain cortical networks at first clinical presentation of psychosis: The OPTiMiSE Study. Schizophr Bull.

[7] Marquand AF, Rezek I, Buitelaar J, Beckmann CF (2016). Understanding heterogeneity in clinical cohorts using normative models: beyond case-control studies. Biol Psychiatry.

[8] Wolfers T, Doan NT, Kaufmann T, Alnæs D, Moberget T, Agartz I (2018). Mapping the heterogeneous phenotype of schizophrenia and bipolar disorder using normative models. JAMA Psychiatry.

[9] Chen J, Liu J, Calhoun VD, Arias-Vasquez A, Zwiers MP, Gupta CN (2014). Exploration of scanning effects in multi-site structural MRI studies. J Neurosci Methods.

[10] Kia SM, Huijsdens H, Rutherford S, Dinga R, Wolfers T, Mennes M, et al. Multi-site normative modeling using hierarchical Bayesian regression. Medical Image Computing and Computer Assisted Intervention – MICCAI 2020. Lecture Notes in Computer Science book series volume 12267, 2021.

[11] Di Forti M, Sallis H, Allegri F, Trotta A, Ferraro L, Stilo SA (2014). Daily use, especially of high-potency cannabis, drives the earlier onset of psychosis in cannabis users. Schizophr Bull.

[12] Palaniyappan L, Marques TR, Taylor H, Handley R, Mondelli V, Bonaccorso S (2013). Cortical folding defects as markers of poor treatment response in first-episode psychosis. JAMA Psychiatry.

[13] Dazzan P, Lawrence AJ, Reinders AATS, Egerton A, Van Haren NEM, Merritt K (2021). Symptom remission and brain cortical networks at first clinical presentation of psychosis: The OPTiMiSE Study. Schizophr Bull.

[14] Andreason NC (2005). Remission in Schizophrenia: Proposed criteria and rationale for consensus. Am J Psychiatry.

[15] Kia SM, Huijsdens H, Rutherford S, de Boer A, Dinga R, Wolfers T (2022). Closing the life-cycle of normative modeling using federated hierarchical Bayesian regression. PLoS One [Internet].

[16] Kia SM, Huijsdens H, Dinga R, Wolfers T, Mennes M, Andreassen OA (2020). Hierarchical Bayesian Regression for Multi-site Normative Modeling of Neuroimaging Data. Lect Notes Comput Sci (including Subser Lect Notes Artif Intell Lect Notes Bioinforma).

[17] Bayer JMM, Dinga R, Kia SM, Kottaram AR, Wolfers T, Lv J (2022). Accommodating site variation in neuroimaging data using normative and hierarchical Bayesian models. Neuroimage.

[18] Gaiser C, Berthet P, Kia SM, Frens MA, Beckmann CF, Muetzel RL. Estimating cortical thickness trajectories in children across different scanners using transfer learning from normative models. Preprint. 2023;1–21. 10.1002/hbm.26565 PMC10839740 38339954

[19] Mann HB, Whitney DR (1947). On a test of whether one of two random variables is stochastically larger than the other author (s): H. B. Mann and D. R. Whitney Published by: Institute of Mathematical Statistics Stable URL. Ann Math Stat.

[20] Benjamini Y, Hochberg Y (1995). Controlling the false discovery rate: A practical and powerful approach to multiple testing. J R Stat Soc Ser B.

[21] Antoniades M, Haas SS, Modabbernia A, Bykowsky O, Frangou S, Borgwardt S (2021). Personalized estimates of brain structural variability in individuals with early Psychosis. Schizophr Bull.

[22] Wolfers T, Rokicki J, Alnæs D, Berthet P, Agartz I, Kia SM (2021). Replicating extensive brain structural heterogeneity in individuals with schizophrenia and bipolar disorder. Hum Brain Mapp.

[23] Leifker FR, Bowie CR, Harvey PD (2009). Determinants of everyday outcomes in schizophrenia: The influences of cognitive impairment, functional capacity, and symptoms. Schizophr Res [Internet].

[24] Gee B, Hodgekins J, Fowler D, Marshall M, Everard L, Lester H (2016). The course of negative symptom in first episode psychosis and the relationship with social recovery. Schizophr Res.

[25] Galderisi S, Rossi A, Rocca P, Bertolino A, Mucci A, Bucci P (2014). The influence of illness-related variables, personal resources and context-related factors on real-life functioning of people with schizophrenia. World Psychiatry.

[26] Dazzan P, Arango C, Fleischacker W, Galderisi S, Glenthøj B, Leucht S (2015). Magnetic resonance imaging and the prediction of outcome in first-episode schizophrenia: A review of current evidence and directions for future research. Schizophr Bull.

[27] Galderisi S, Rucci P, Kirkpatrick B, Mucci A, Gibertoni D, Rocca P (2018). Interplay among psychopathologic variables, personal resources, context-related factors, and real-life functioning in individuals with schizophrenia a network analysis. JAMA Psychiatry.

[28] Kirkpatrick B, Fenton WS, Carpenter WT, Marder SR (2006). The NIMH-MATRICS consensus statement on negative symptoms. Schizophr Bull.

[29] Austin SF, Mors O, Budtz-Jørgensen E, Secher RG, Hjorthøj CR, Bertelsen M (2015). Long-term trajectories of positive and negative symptoms in first episode psychosis: A 10year follow-up study in the OPUS cohort. Schizophr Res [Internet].

[30] Schennach R, Meyer S, Seemüller F, Jäger M, Schmauss M, Laux G (2012). Response trajectories in “real-world” naturalistically treated schizophrenia patients. Schizophr Res.

[31] Deakin B, Suckling J, Barnes TRE, Byrne K, Chaudhry IB, Dazzan P (2018). The benefit of minocycline on negative symptoms of schizophrenia in patients with recent-onset psychosis (BeneMin): a randomised, double-blind, placebo-controlled trial. Lancet Psychiatry.

[32] Dazzan P, Lappin JM, Heslin M, Donoghue K, Lomas B, Reininghaus U (2019). Symptom remission at 12-weeks strongly predicts long-term recovery from the first episode of psychosis. Psychol Med.

