# Supplementary material for: Extreme deviations from the normative model reveal cortical heterogeneity and associations with negative symptom severity in first-episode psychosis from the OPTiMiSE and GAP studies
Source: Transl Psychiatry. 2023 Dec 2;13:373. doi: 10.1038/s41398-023-02661-6 (PMC10693627; doi:10.1038/s41398-023-02661-6)
Supplement: Supplementary file 1 — Supplementary material [file 41398_2023_2661_MOESM1_ESM.docx]

**Table S1**: Site-specific MR Scanner Details

Field

| Scanning Site | Scanner  Manufacturer | Scanner Model | Strength (T) | Software Version |
| --- | --- | --- | --- | --- |
| Czech Republic (CZ) | SIEMENS | TrioTim | 3 | syngo MR B17 |
| Denmark (DK) | Philips Medical Systems | Achieva | 3 | 3.2.1\3.2.1.1 |
| Israel (IL) | GE MEDICAL SYSTEMS | Signa HDxt | 3 | 15\LX\MR  Software release:15.0_M4A_0947.a |
| Italy (IT) | SIEMENS | TrioTim | 3 | syngo MR B17 |
| Netherlands (NL) | Philips Medical Systems | Achieva | 3 | 3.2.3\3.2.3.1 |
| Spain (ES) | GE MEDICAL SYSTEMS | Signa HDxt | 3 | 24\LX\MR  Software release:HD16.0_V01_1108.b |
| United Kingdom (UK) | GE MEDICAL SYSTEMS | Signa HDx | 3 | 14\LX\MR Software release:14.0_M5_0737.f |

**Table S2:** Site-specific MR Sequence Details

| **Scanning** | **Scan** | **Slice**  **Thicknes s** | **Slice Gap** | **TR** | **TE** | **TI** | **FA** |  | **Acquisition** | **FoV** |
| --- | --- | --- | --- | --- | --- | --- | --- | --- | --- | --- |
| **Site** | **Sequence Plane 3D / 2D** | **(mm)** | **(mm)** | **(ms)** | **(ms)** | **(ms)** | **(°)** | **NEX** | **Matrix** | **(mm)** |
| Czech | MPRAGE Sagittal 3D | 1.2 | 0 | 230 | 2.98 | 900 | 9 | 1 | 256x240 | 256x24 |
| Republic (CZ) |  |  |  | 0 |  |  |  |  |  | 0 |
| Denmark | MPRAGE Sagittal 3D | 1.2 | 0 | 6.69 | 3.12 | - | 9 | 1 | 256x256 | 256x25 |
| (DK) |  |  |  |  | 2 |  |  |  |  | 6 |
| Israel | IR-SPGR Sagittal 3D | 1.2 | 0 | 7.00 | 2.85 | 400 | 11 | 1 | 256x256 | 256x25 |
| (IL) |  |  |  |  | 6 |  |  |  |  | 6 |
| Italy | MPRAGE Sagittal 3D | 1.2 | 0 | 230 | 2.98 | 900 | 9 | 1 | 256x240 | 256x24 |
| (IT) |  |  |  | 0 |  |  |  |  |  | 0 |
| Netherlands | MPRAGE Sagittal 3D | 1.2 | 0 | 6.77 | 3.13 | - | 9 | 1 | 256x256 | 256x25 |
| (NL) |  |  |  |  | 3 |  |  |  |  | 6 |
| Spain | IR-SPGR Sagittal 3D | 1.2 | 0 | 6.58 | 2.80 | 400 | 11 | 1 | 256x256 | 256x25 |
| (ES) |  |  |  |  | 4 |  |  |  |  | 6 |
| United | IR-SPGR Sagittal 3D | 1.2 | 0 | 6.89 | 2.84 | 400 | 11 | 1 | 256x256 | 256x25 |
| Kingdom (UK) |  |  |  |  | 8 |  |  |  |  | 6 |

)

| **Table S3: Site-wise demographic and clinical** | **test data** |  | | | | | | | | | | | | | | | | | | | |
| --- | --- | --- | --- | --- | --- | --- | --- | --- | --- | --- | --- | --- | --- | --- | --- | --- | --- | --- | --- | --- | --- |
|  |  | **GAP** |  |  | **UK** |  |  | **IT** |  |  | **NL** |  |  | **IL** |  |  | **ES** |  |  | **DK** |  |
|  | Patients |  | Controls | Patients |  | Controls | Patients |  | Controls | Patients |  | Controls | Patients |  | Controls | Patients |  | Controls | Patients |  | Controls |
|  | N = 123 |  | N = 94 | N = 22 |  | N = 34 | N = 25 |  | N = 14 | N = 12 |  | N = 9 | N = 47 |  | N = 19 | N = 36 |  | N = 23 | N = 30 |  | N = 15 |
| Age, mean years (s.d.) | 28(7.28) |  | 27(7.36) | 27(6.09) |  | 25(4.19) | 28(8.40) |  | 27(6.84) | 25(5.37) |  | 22(1.83) | 24(5.47) |  | 25(5.37) | 25(5.46) |  | 27(6.04) | 22(3.27) |  | 22(3.15) |
| Sex, N male, (%) | 37(30) |  | 37(39) | 16 (59) |  | 26 (77) | 7 (28) |  | 4 (29) | 11 (92) |  | 4 (44) | 40 (85) |  | 12 (63) | 26 (72) |  | 17 (74) | 15 (50) |  | 9 (60) |
| Ethnicity |  |  |  |  |  |  |  |  |  |  |  |  |  |  |  |  |  |  |  |  |  |
| N white (%) | 50(41) |  | 50(53) | 10(45) |  | 0 | 22(88) |  | 14(100) | 10(83) |  | 0 | 45(96) |  | 19(100) | 27(75) |  | 21(91) | 28(93) |  | 15(100) |
| N black (%) | 44(36) |  | 32(34) | 5(23) |  | 0 | 2(8) |  | 0 | 1(8.5) |  | 0 | 2(4) |  | 0 | 2(5) |  | 0 | 0 |  | 0 |
| N Asian (%) | 19(15) |  | 1(1) | 4(18) |  | 0 | 1() |  | 0 | 1(8.5) |  | 0 | 0 |  | 0 | 0 |  | 0 | 2(7) |  | 0 |
| N other (%) | 10(8) |  | 11(12) | 3(14) |  | 0 | 0(4) |  | 0 | 0 |  | 0 | 0 |  | 0 | 6(17) |  | 2(9) | 0 |  | 0 |
| N unknown (%) | 0(0) |  | 0(0) | 0 |  | 34(100) | 0 |  | 0 | 0 |  | 9(100) | 0 |  | 0 | 1(3) |  | 0 | 0 |  | 0 |
| **Clinical follow-up** |  |  |  |  |  |  |  |  |  |  |  |  |  |  |  |  |  |  |  |  |  |
| Assessments taken at weeks median, (min, max)  Number of longitudinal assessments | 9(0,57)  2(1,3) |  | -  - | 4(0,86)  7(1,9) |  | -  - | 5(0,76  ) 8(2,12 |  | -  - | 3(0,76)  4(4,9) |  | -  - | 7(0,95)  8(3,15) |  | -  - | 4(0,94)  6(2,10) |  | -  - | 4(0,68)  4(1, 8) |  | -  - |
| median, (min, max)  PANSS baseline, N | 96 |  | - | 19 |  | - | )  25 |  | - | 13 |  | - | 47 |  | - | 34 |  | - | 30 |  | - |
| Positive median, (min, max) | 14(7,32) |  | - | 17(12, |  | - | 18(9,2 |  | - | 13(12,24) |  | - | 16(7,31) |  | - | 17(7,28) |  | - | 22(11,35) |  | - |
|  |  |  |  | 28) |  |  | 8) |  |  |  |  |  |  |  |  |  |  |  |  |  |  |
| Negative median, (min, max) | 15(7,29) |  | - | 14(8,23) |  | - | 15(7,3 |  | - | 12(7,27) |  | - | 18(7,31) |  | - | 16(9,33) |  | - | 22(9,33) |  | - |
|  |  |  |  |  |  |  | 6) |  |  |  |  |  |  |  |  |  |  |  |  |  |  |
| General median, (min, max) | 28(16,52 |  | - | 32(23,49) |  | - | 37(25, |  | - | 33(25,50) |  | - | 33(17,53) |  | - | 35(21,49) |  | - | 43(24,59) |  | - |
|  |  |  |  |  |  |  | 57) |  |  |  |  |  |  |  |  |  |  |  |  |  |  |

**Table S4:** Results from linear mixed effects model predicting medium term negative symptoms from weeks and cortical thickness z-scores

| **Rank** | **Threshold** | **p-value** | **Region** | **Benjamin_Hochberg p-value** |
| --- | --- | --- | --- | --- |
| **1** | **0.05** | **0.00007** | **L_G_postcentral** | **0.000336** |
| **2** | **0.05** | **0.00015** | **L_G_pariet_inf.Supramar** | **0.000671** |
| **3** | **0.05** | **0.00019** | **L_G_occipital_sup** | **0.001007** |
| **4** | **0.05** | **0.00036** | **L_G.S_subcentral** | **0.001342** |
| **5** | **0.05** | **0.00080** | **L_S_postcentral** | **0.001678** |
| **6** | **0.05** | **0.00097** | **mean_thickness** | **0.002013** |
| **7** | **0.05** | **0.00135** | **R_G.S_paracentral** | **0.002349** |
| **8** | **0.05** | **0.00142** | **R_S_occipital_ant** | **0.002685** |
| **9** | **0.05** | **0.00154** | **R_G_temp_sup.Plan_tempo** | **0.00302** |
| **10** | **0.05** | **0.00186** | **L_S_temporal_inf** | **0.003356** |
| **11** | **0.05** | **0.00219** | **L_S_intrapariet.P_trans** | **0.003691** |
| **12** | **0.05** | **0.00304** | **L_G_temp_sup.Lateral** | **0.004027** |
| **13** | **0.05** | **0.00393** | **L_S_precentral.sup.part** | **0.004362** |
| 14 | 0.05 | 0.00499 | R_G_orbital | 0.004698 |
| 15 | 0.05 | 0.00584 | L_S_central | 0.005034 |
| 16 | 0.05 | 0.00642 | L_G_temp_sup.Plan_tempo | 0.005369 |
| 17 | 0.05 | 0.00819 | L_S_oc_sup.transversal | 0.005705 |
| 18 | 0.05 | 0.00825 | L_S_temporal_sup | 0.006040 |
| 19 | 0.05 | 0.00877 | L_G_pariet_inf.Angular | 0.006376 |
| 20 | 0.05 | 0.00887 | R_S_oc.temp_lat | 0.006711 |
| 21 | 0.05 | 0.00997 | R_G_occipital_sup | 0.007047 |
| 22 | 0.05 | 0.01000 | L_Pole_occipital | 0.007383 |
| 23 | 0.05 | 0.01290 | L_S_circular_insula_inf | 0.007718 |
| 24 | 0.05 | 0.01310 | R_G.S_cingul.Mid.Post | 0.008054 |
| 25 | 0.05 | 0.01310 | R_S_central | 0.008389 |
| 26 | 0.05 | 0.01370 | R_S_temporal_transverse | 0.008725 |
| 27 | 0.05 | 0.01570 | L_S_front_inf | 0.009060 |
| 28 | 0.05 | 0.01580 | L_G_orbital | 0.009396 |
| 29 | 0.05 | 0.01630 | R_G_parietal_sup | 0.009732 |
| 30 | 0.05 | 0.01790 | R_G_postcentral | 0.010067 |
| 31 | 0.05 | 0.01800 | R_G.S_occipital_inf | 0.010403 |
| 32 | 0.05 | 0.01930 | R_S_subparietal | 0.010738 |
| 33 | 0.05 | 0.02020 | L_G_precentral | 0.011074 |
| 34 | 0.05 | 0.02340 | L_S_front_middle | 0.011409 |
| 35 | 0.05 | 0.02370 | R_G_occipital_middle | 0.011745 |
| 36 | 0.05 | 0.02410 | L_G_temporal_middle | 0.012081 |
| 37 | 0.05 | 0.02470 | R_S_collat_transv_ant | 0.012416 |
| 38 | 0.05 | 0.02600 | L_G_cingul.Post.ventral | 0.012752 |
| 39 | 0.05 | 0.02650 | L_S_oc_middle.Lunatus | 0.013087 |
| 40 | 0.05 | 0.02840 | R_G_temporal_middle | 0.013423 |
| 41 | 0.05 | 0.03000 | R_G_cuneus | 0.013758 |
| 42 | 0.05 | 0.03180 | R_S_oc_sup.transversal | 0.014094 |
| 43 | 0.05 | 0.03190 | R_G_temp_sup.Lateral | 0.014430 |
| 44 | 0.05 | 0.03420 | R_G_temporal_inf | 0.014765 |
| 45 | 0.05 | 0.04020 | L_S_temporal_transverse | 0.015101 |
| 46 | 0.05 | 0.04190 | R_G_temp_sup.Plan_polar | 0.015436 |
| 47 | 0.05 | 0.04520 | L_G.S_paracentral | 0.015772 |

| 48 | 0.05 | 0.04850 | R_Lat_Fis.post | 0.016107 |
| --- | --- | --- | --- | --- |
| 49 | 0.05 | 0.05260 | L_G_Ins_lg.S_cent_ins | 0.016443 |
| 50 | 0.05 | 0.05280 | L_G_rectus | 0.016779 |
| 51 | 0.05 | 0.05290 | L_S_subparietal | 0.017114 |
| 52 | 0.05 | 0.05520 | R_S_temporal_inf | 0.017450 |
| 53 | 0.05 | 0.05640 | L_G_cuneus | 0.017785 |
| 54 | 0.05 | 0.05640 | L_S_front_sup | 0.018121 |
| 55 | 0.05 | 0.05910 | L_S_cingul.Marginalis | 0.018456 |
| 56 | 0.05 | 0.05940 | L_G_cingul.Post.dorsal | 0.018792 |
| 57 | 0.05 | 0.06000 | R_S_suborbital | 0.019128 |
| 58 | 0.05 | 0.06600 | R_G_Ins_lg.S_cent_ins | 0.019463 |
| 59 | 0.05 | 0.06610 | R_S_temporal_sup | 0.019799 |
| 60 | 0.05 | 0.06690 | R_G_pariet_inf.Angular | 0.020134 |
| 61 | 0.05 | 0.07090 | R_G_precuneus | 0.020470 |
| 62 | 0.05 | 0.07180 | R_S_precentral.sup.part | 0.020805 |
| 63 | 0.05 | 0.07250 | R_G_front_sup | 0.021141 |
| 64 | 0.05 | 0.07660 | L_G_front_inf.Opercular | 0.021477 |
| 65 | 0.05 | 0.07840 | L_Lat_Fis.post | 0.021812 |
| 66 | 0.05 | 0.07860 | R_S_parieto_occipital | 0.022148 |
| 67 | 0.05 | 0.07860 | R_S_postcentral | 0.022483 |
| 68 | 0.05 | 0.08220 | R_S_interm_prim.Jensen | 0.022819 |
| 69 | 0.05 | 0.08300 | L_G_precuneus | 0.023154 |
| 70 | 0.05 | 0.08350 | L_G_front_middle | 0.023490 |
| 71 | 0.05 | 0.08580 | L_S_occipital_ant | 0.023826 |
| 72 | 0.05 | 0.08810 | R_S_intrapariet.P_trans | 0.024161 |
| 73 | 0.05 | 0.08930 | L_Pole_temporal | 0.024497 |
| 74 | 0.05 | 0.09140 | L_S_precentral.inf.part | 0.024832 |
| 75 | 0.05 | 0.09150 | R_S_calcarine | 0.025168 |
| 76 | 0.05 | 0.09620 | R_G_cingul.Post.dorsal | 0.025503 |
| 77 | 0.05 | 0.09860 | R_G_cingul.Post.ventral | 0.025839 |
| 78 | 0.05 | 0.10100 | L_G_parietal_sup | 0.026174 |
| 79 | 0.05 | 0.10700 | L_S_orbital_lateral | 0.026510 |
| 80 | 0.05 | 0.10800 | L_S_collat_transv_post | 0.026846 |
| 81 | 0.05 | 0.11800 | R_S_oc_middle.Lunatus | 0.027181 |
| 82 | 0.05 | 0.12400 | L_G_temp_sup.G_T_transv | 0.027517 |
| 83 | 0.05 | 0.13000 | L_G_temp_sup.Plan_polar | 0.027852 |
| 84 | 0.05 | 0.13900 | R_S_cingul.Marginalis | 0.028188 |
| 85 | 0.05 | 0.15000 | L_S_oc.temp_med.Lingual | 0.028523 |
| 86 | 0.05 | 0.15500 | R_S_orbital_med.olfact | 0.028859 |
| 87 | 0.05 | 0.15600 | L_G.S_occipital_inf | 0.029195 |
| 88 | 0.05 | 0.16500 | R_Pole_temporal | 0.029530 |
| 89 | 0.05 | 0.16600 | R_S_orbital.H_Shaped | 0.029866 |
| 90 | 0.05 | 0.17000 | L_G.S_cingul.Mid.Ant | 0.030201 |
| 91 | 0.05 | 0.17400 | L_Lat_Fis.ant.Vertical | 0.030537 |
| 92 | 0.05 | 0.17500 | R_G_precentral | 0.030872 |
| 93 | 0.05 | 0.18300 | R_G_oc.temp_lat.fusifor | 0.031208 |
| 94 | 0.05 | 0.19800 | L_G.S_frontomargin | 0.031544 |
| 95 | 0.05 | 0.19900 | R_S_front_sup | 0.031879 |
| 96 | 0.05 | 0.20400 | R_G_front_middle | 0.032215 |
| 97 | 0.05 | 0.21200 | R_Lat_Fis.ant.Vertical | 0.032550 |
| 98 | 0.05 | 0.21500 | R_G_front_inf.Triangul | 0.032886 |

| 99 | 0.05 | 0.22900 | L_G_front_sup | 0.033221 |
| --- | --- | --- | --- | --- |
| 100 | 0.05 | 0.23000 | L_S_orbital.H_Shaped | 0.033557 |
| 101 | 0.05 | 0.23300 | R_S_pericallosal | 0.033893 |
| 102 | 0.05 | 0.23900 | R_G.S_subcentral | 0.034228 |
| 103 | 0.05 | 0.24000 | L_S_pericallosal | 0.034564 |
| 104 | 0.05 | 0.24900 | L_G_front_inf.Triangul | 0.034899 |
| 105 | 0.05 | 0.24900 | L_S_collat_transv_ant | 0.035235 |
| 106 | 0.05 | 0.25100 | R_G_rectus | 0.035570 |
| 107 | 0.05 | 0.25900 | L_G.S_transv_frontopol | 0.035906 |
| 108 | 0.05 | 0.26000 | R_Lat_Fis.ant.Horizont | 0.036242 |
| 109 | 0.05 | 0.27000 | L_S_suborbital | 0.036577 |
| 110 | 0.05 | 0.27600 | R_G_pariet_inf.Supramar | 0.036913 |
| 111 | 0.05 | 0.27900 | R_S_front_middle | 0.037248 |
| 112 | 0.05 | 0.28000 | L_G_subcallosal | 0.037584 |
| 113 | 0.05 | 0.28400 | L_G_oc.temp_med.Lingual | 0.037919 |
| 114 | 0.05 | 0.28400 | R_G_subcallosal | 0.038255 |
| 115 | 0.05 | 0.28700 | R_Pole_occipital | 0.038591 |
| 116 | 0.05 | 0.29800 | L_G_temporal_inf | 0.038926 |
| 117 | 0.05 | 0.31700 | L_S_circular_insula_ant | 0.039262 |
| 118 | 0.05 | 0.32600 | L_G_occipital_middl | 0.039597 |
| 119 | 0.05 | 0.35100 | R_S_front_inf | 0.039933 |
| 120 | 0.05 | 0.35800 | L_S_circular_insula_sup | 0.040268 |
| 121 | 0.05 | 0.36800 | L_G_front_inf.Orbital | 0.040604 |
| 122 | 0.05 | 0.37300 | L_S_oc.temp_lat | 0.040940 |
| 123 | 0.05 | 0.38400 | L_S_calcarine | 0.041275 |
| 124 | 0.05 | 0.38600 | L_S_parieto_occipital | 0.041611 |
| 125 | 0.05 | 0.39000 | R_G_temp_sup.G_T_transv | 0.041946 |
| 126 | 0.05 | 0.39700 | R_G.S_frontomargin | 0.042282 |
| 127 | 0.05 | 0.40800 | R_G_oc.temp_med.Lingual | 0.042617 |
| 128 | 0.05 | 0.41800 | L_S_interm_prim.Jensen | 0.042953 |
| 129 | 0.05 | 0.41900 | L_G_insular_short | 0.043289 |
| 130 | 0.05 | 0.42700 | L_G.S_cingul.Mid.Post | 0.043624 |
| 131 | 0.05 | 0.50500 | L_G_oc.temp_lat.fusifor | 0.043960 |
| 132 | 0.05 | 0.58800 | R_G.S_transv_frontopol | 0.044295 |
| 133 | 0.05 | 0.64000 | R_S_circular_insula_inf | 0.044631 |
| 134 | 0.05 | 0.67000 | L_S_orbital_med.olfact | 0.044966 |
| 135 | 0.05 | 0.69900 | R_S_circular_insula_ant | 0.045302 |
| 136 | 0.05 | 0.72000 | R_S_precentral.inf.part | 0.045638 |
| 137 | 0.05 | 0.73400 | R_S_oc.temp_med.Lingual | 0.045973 |
| 138 | 0.05 | 0.74200 | R_G.S_cingul.Mid.Ant | 0.046309 |
| 139 | 0.05 | 0.76100 | R_G.S_cingul.Ant | 0.046644 |
| 140 | 0.05 | 0.77700 | L_G.S_cingul.Ant | 0.046980 |
| 141 | 0.05 | 0.79600 | L_G_oc.temp_med.Parahip | 0.047315 |
| 142 | 0.05 | 0.81600 | R_G_oc.temp_med.Parahip | 0.047651 |
| 143 | 0.05 | 0.83900 | R_S_circular_insula_sup | 0.047987 |
| 144 | 0.05 | 0.86300 | R_G_insular_short | 0.048322 |
| 145 | 0.05 | 0.89800 | R_G_front_inf.Opercular | 0.048658 |
| 146 | 0.05 | 0.91600 | R_S_orbital_lateral | 0.048993 |
| 147 | 0.05 | 0.92200 | R_S_collat_transv_post | 0.049329 |
| 148 | 0.05 | 0.93300 | R_G_front_inf.Orbital | 0.049664 |
| 149 | 0.05 | 0.94400 | L_Lat_Fis.ant.Horizont | 0.050000 |

**Table S5:** Results from linear mixed effects model predicting medium term positive symptoms from weeks and cortical thickness z-scores

| **Rank** | **Threshold** | **p-value** | **Region** | **Benjamin_Hochberg p-value** |
| --- | --- | --- | --- | --- |
| 1 | 0.05 | 0.0087 | R_G_Ins_lg.S_cent_ins | 0.000336 |
| 2 | 0.05 | 0.0108 | L_S_temporal_inf | 0.000671 |
| 3 | 0.05 | 0.0165 | R_S_temporal_transverse | 0.001007 |
| 4 | 0.05 | 0.0226 | L_G_temp_sup.G_T_transv | 0.001342 |
| 5 | 0.05 | 0.0279 | R_Pole_occipital | 0.001678 |
| 6 | 0.05 | 0.0413 | R_G_orbital | 0.002013 |
| 7 | 0.05 | 0.0416 | R_S_temporal_inf | 0.002349 |
| 8 | 0.05 | 0.0553 | R_G.S_transv_frontopol | 0.002685 |
| 9 | 0.05 | 0.0555 | R_G_occipital_middle | 0.003020 |
| 10 | 0.05 | 0.0645 | L_S_front_inf | 0.003356 |
| 11 | 0.05 | 0.0677 | R_G.S_subcentral | 0.003691 |
| 12 | 0.05 | 0.0730 | R_G_temp_sup.G_T_transv | 0.004027 |
| 13 | 0.05 | 0.0855 | R_S_interm_prim.Jensen | 0.004362 |
| 14 | 0.05 | 0.1140 | L_G_occipital_sup | 0.004698 |
| 15 | 0.05 | 0.1200 | R_G.S_cingul.Ant | 0.005034 |
| 16 | 0.05 | 0.1210 | R_S_parieto_occipital | 0.005369 |
| 17 | 0.05 | 0.1220 | R_S_precentral.sup.part | 0.005705 |
| 18 | 0.05 | 0.1230 | R_S_orbital_med.olfact | 0.006040 |
| 19 | 0.05 | 0.1250 | R_S_front_inf | 0.006376 |
| 20 | 0.05 | 0.1430 | L_G_temp_sup.Plan_polar | 0.006711 |
| 21 | 0.05 | 0.1480 | L_S_oc.temp_med.Lingual | 0.007047 |
| 22 | 0.05 | 0.1600 | L_S_oc_sup.transversal | 0.007383 |
| 23 | 0.05 | 0.1840 | R_S_oc_sup.transversal | 0.007718 |
| 24 | 0.05 | 0.1880 | L_G_front_inf.Orbital | 0.008054 |
| 25 | 0.05 | 0.1890 | L_G_temporal_middle | 0.008389 |
| 26 | 0.05 | 0.1900 | L_S_central | 0.008725 |
| 27 | 0.05 | 0.1940 | L_S_intrapariet.P_trans | 0.009060 |
| 28 | 0.05 | 0.1940 | R_Pole_temporal | 0.009396 |
| 29 | 0.05 | 0.1960 | L_S_interm_prim.Jensen | 0.009732 |
| 30 | 0.05 | 0.1960 | L_S_pericallosal | 0.010067 |
| 31 | 0.05 | 0.2010 | R_S_occipital_ant | 0.010403 |
| 32 | 0.05 | 0.2020 | R_Lat_Fis.ant.Horizont | 0.010738 |
| 33 | 0.05 | 0.2160 | R_S_collat_transv_post | 0.011074 |
| 34 | 0.05 | 0.2230 | R_S_central | 0.011409 |
| 35 | 0.05 | 0.2250 | L_S_subparietal | 0.011745 |
| 36 | 0.05 | 0.2270 | R_G_rectus | 0.012081 |
| 37 | 0.05 | 0.2580 | L_G_pariet_inf.Angular | 0.012416 |
| 38 | 0.05 | 0.2610 | L_S_occipital_ant | 0.012752 |
| 39 | 0.05 | 0.2650 | R_G_front_sup | 0.013087 |
| 40 | 0.05 | 0.2680 | R_G.S_occipital_inf | 0.013423 |
| 41 | 0.05 | 0.2680 | R_S_postcentral | 0.013758 |
| 42 | 0.05 | 0.2690 | L_Lat_Fis.ant.Vertical | 0.014094 |
| 43 | 0.05 | 0.2730 | R_G.S_cingul.Mid.Ant | 0.014430 |
| 44 | 0.05 | 0.2820 | L_G_precentral | 0.014765 |
| 45 | 0.05 | 0.2840 | R_S_oc_middle.Lunatus | 0.015101 |
| 46 | 0.05 | 0.2940 | L_S_orbital_med.olfact | 0.015436 |
| 47 | 0.05 | 0.2980 | L_Pole_occipital | 0.015772 |
| 48 | 0.05 | 0.3050 | L_S_precentral.inf.part | 0.016107 |

| 49 | 0.05 | 0.3170 | L_G_front_sup | 0.016443 |
| --- | --- | --- | --- | --- |
| 50 | 0.05 | 0.3180 | L_G_Ins_lg.S_cent_ins | 0.016779 |
| 51 | 0.05 | 0.3340 | L_G_rectus | 0.017114 |
| 52 | 0.05 | 0.3410 | R_G_front_inf.Triangul | 0.017450 |
| 53 | 0.05 | 0.3430 | L_S_front_middle | 0.017785 |
| 54 | 0.05 | 0.3470 | L_S_circular_insula_inf | 0.018121 |
| 55 | 0.05 | 0.3630 | R_G_subcallosal | 0.018456 |
| 56 | 0.05 | 0.3660 | L_G_front_middle | 0.018792 |
| 57 | 0.05 | 0.4110 | L_G.S_occipital_inf | 0.019128 |
| 58 | 0.05 | 0.4150 | R_G_temp_sup.Lateral | 0.019463 |
| 59 | 0.05 | 0.4160 | L_G.S_paracentral | 0.019799 |
| 60 | 0.05 | 0.4200 | R_G_cingul.Post.ventral | 0.020134 |
| 61 | 0.05 | 0.4200 | R_S_suborbital | 0.020470 |
| 62 | 0.05 | 0.4220 | R_G_temp_sup.Plan_tempo | 0.020805 |
| 63 | 0.05 | 0.4490 | R_S_intrapariet.P_trans | 0.021141 |
| 64 | 0.05 | 0.4520 | R_G_insular_short | 0.021477 |
| 65 | 0.05 | 0.4530 | L_S_postcentral | 0.021812 |
| 66 | 0.05 | 0.4530 | R_G_temporal_middle | 0.022148 |
| 67 | 0.05 | 0.4550 | L_S_temporal_sup | 0.022483 |
| 68 | 0.05 | 0.4590 | R_S_orbital_lateral | 0.022819 |
| 69 | 0.05 | 0.4600 | L_S_oc_middle.Lunatus | 0.023154 |
| 70 | 0.05 | 0.4640 | L_G_pariet_inf.Supramar | 0.023490 |
| 71 | 0.05 | 0.4700 | R_S_oc.temp_lat | 0.023826 |
| 72 | 0.05 | 0.4750 | R_S_temporal_sup | 0.024161 |
| 73 | 0.05 | 0.4780 | L_G.S_transv_frontopol | 0.024497 |
| 74 | 0.05 | 0.4800 | L_S_parieto_occipital | 0.024832 |
| 75 | 0.05 | 0.4990 | L_S_calcarine | 0.025168 |
| 76 | 0.05 | 0.5060 | L_G_oc.temp_med.Lingual | 0.025503 |
| 77 | 0.05 | 0.5060 | L_S_front_sup | 0.025839 |
| 78 | 0.05 | 0.5130 | R_G_temp_sup.Plan_polar | 0.026174 |
| 79 | 0.05 | 0.5220 | R_S_orbital.H_Shaped | 0.026510 |
| 80 | 0.05 | 0.5330 | mean_thickness | 0.026846 |
| 81 | 0.05 | 0.5390 | L_G.S_cingul.Mid.Post | 0.027181 |
| 82 | 0.05 | 0.5440 | R_G_occipital_sup | 0.027517 |
| 83 | 0.05 | 0.5460 | L_G.S_cingul.Mid.Ant | 0.027852 |
| 84 | 0.05 | 0.5560 | L_Lat_Fis.post | 0.028188 |
| 85 | 0.05 | 0.5590 | L_G.S_cingul.Ant | 0.028523 |
| 86 | 0.05 | 0.5630 | R_G_cuneus | 0.028859 |
| 87 | 0.05 | 0.5710 | L_G_oc.temp_med.Parahip | 0.029195 |
| 88 | 0.05 | 0.5730 | R_G.S_cingul.Mid.Post | 0.029530 |
| 89 | 0.05 | 0.5760 | R_G_postcentral | 0.029866 |
| 90 | 0.05 | 0.5840 | R_G_precuneus | 0.030201 |
| 91 | 0.05 | 0.5870 | L_G_cingul.Post.ventral | 0.030537 |
| 92 | 0.05 | 0.5910 | R_G_cingul.Post.dorsal | 0.030872 |
| 93 | 0.05 | 0.5940 | R_S_oc.temp_med.Lingual | 0.031208 |
| 94 | 0.05 | 0.6110 | R_G_oc.temp_med.Lingual | 0.031544 |
| 95 | 0.05 | 0.6130 | R_G_oc.temp_med.Parahip | 0.031879 |
| 96 | 0.05 | 0.6150 | L_S_oc.temp_lat | 0.032215 |
| 97 | 0.05 | 0.6190 | L_G_cuneus | 0.032550 |
| 98 | 0.05 | 0.6270 | L_G.S_subcentral | 0.032886 |
| 99 | 0.05 | 0.6360 | L_G_front_inf.Opercular | 0.033221 |

| 100 | 0.05 | 0.6410 | R_G.S_paracentral | 0.033557 |
| --- | --- | --- | --- | --- |
| 101 | 0.05 | 0.6430 | L_S_temporal_transverse | 0.033893 |
| 102 | 0.05 | 0.6440 | R_S_front_middle | 0.034228 |
| 103 | 0.05 | 0.6470 | R_S_collat_transv_ant | 0.034564 |
| 104 | 0.05 | 0.6510 | R_S_circular_insula_inf | 0.034899 |
| 105 | 0.05 | 0.6580 | L_G_parietal_sup | 0.035235 |
| 106 | 0.05 | 0.6640 | L_S_collat_transv_ant | 0.035570 |
| 107 | 0.05 | 0.6700 | R_G_oc.temp_lat.fusifor | 0.035906 |
| 108 | 0.05 | 0.6850 | R_S_calcarine | 0.036242 |
| 109 | 0.05 | 0.6900 | L_G_precuneus | 0.036577 |
| 110 | 0.05 | 0.6950 | R_Lat_Fis.post | 0.036913 |
| 111 | 0.05 | 0.7020 | R_S_circular_insula_ant | 0.037248 |
| 112 | 0.05 | 0.7260 | L_S_circular_insula_ant | 0.037584 |
| 113 | 0.05 | 0.7290 | L_G.S_frontomargin | 0.037919 |
| 114 | 0.05 | 0.7380 | L_G_temp_sup.Plan_tempo | 0.038255 |
| 115 | 0.05 | 0.7540 | R_Lat_Fis.ant.Vertical | 0.038591 |
| 116 | 0.05 | 0.7620 | L_G_cingul.Post.dorsal | 0.038926 |
| 117 | 0.05 | 0.7660 | R_S_precentral.inf.part | 0.039262 |
| 118 | 0.05 | 0.7810 | L_G_temporal_inf | 0.039597 |
| 119 | 0.05 | 0.7820 | L_S_suborbital | 0.039933 |
| 120 | 0.05 | 0.7870 | L_S_collat_transv_post | 0.040268 |
| 121 | 0.05 | 0.7940 | R_G.S_frontomargin | 0.040604 |
| 122 | 0.05 | 0.8160 | R_S_pericallosal | 0.040940 |
| 123 | 0.05 | 0.8170 | L_G_oc.temp_lat.fusifor | 0.041275 |
| 124 | 0.05 | 0.8230 | L_G_orbital | 0.041611 |
| 125 | 0.05 | 0.8280 | L_S_orbital_lateral | 0.041946 |
| 126 | 0.05 | 0.8320 | R_G_pariet_inf.Supramar | 0.042282 |
| 127 | 0.05 | 0.8340 | R_S_front_sup | 0.042617 |
| 128 | 0.05 | 0.8400 | L_G_postcentral | 0.042953 |
| 129 | 0.05 | 0.8490 | L_S_precentral.sup.part | 0.043289 |
| 130 | 0.05 | 0.8670 | L_S_orbital.H_Shaped | 0.043624 |
| 131 | 0.05 | 0.8780 | R_G_temporal_inf | 0.043960 |
| 132 | 0.05 | 0.8850 | R_G_pariet_inf.Angular | 0.044295 |
| 133 | 0.05 | 0.9020 | L_Pole_temporal | 0.044631 |
| 134 | 0.05 | 0.9160 | R_S_subparietal | 0.044966 |
| 135 | 0.05 | 0.9180 | R_G_front_middle | 0.045302 |
| 136 | 0.05 | 0.9230 | L_S_cingul.Marginalis | 0.045638 |
| 137 | 0.05 | 0.9270 | L_G_front_inf.Triangul | 0.045973 |
| 138 | 0.05 | 0.9350 | R_G_front_inf.Opercular | 0.046309 |
| 139 | 0.05 | 0.9360 | R_G_front_inf.Orbital | 0.046644 |
| 140 | 0.05 | 0.9660 | R_G_parietal_sup | 0.046980 |
| 141 | 0.05 | 0.9660 | R_S_circular_insula_sup | 0.047315 |
| 142 | 0.05 | 0.9670 | L_G_subcallosal | 0.047651 |
| 143 | 0.05 | 0.9680 | R_G_precentral | 0.047987 |
| 144 | 0.05 | 0.9760 | L_G_temp_sup.Lateral | 0.048322 |
| 145 | 0.05 | 0.9770 | L_Lat_Fis.ant.Horizont | 0.048658 |
| 146 | 0.05 | 0.9800 | L_S_circular_insula_sup | 0.048993 |
| 147 | 0.05 | 0.9900 | R_S_cingul.Marginalis | 0.049329 |
| 148 | 0.05 | 0.9930 | L_G_occipital_middle | 0.049664 |
| 149 | 0.05 | 0.9960 | L_G_insular_short | 0.050000 |

**Table S6:** Results from linear mixed effects model predicting medium term general symptoms from weeks and cortical thickness z-scores

| **Rank** | **Threshold** | **p-value** | **Region** | **Benjamin_Hochberg p-value** |
| --- | --- | --- | --- | --- |
| 1 | 0.05 | 0.0067 | R_G_occipital_middle | 0.000336 |
| 2 | 0.05 | 0.0083 | R_G_subcallosal | 0.000671 |
| 3 | 0.05 | 0.0192 | R_S_central | 0.001007 |
| 4 | 0.05 | 0.0214 | L_G_occipital_sup | 0.001342 |
| 5 | 0.05 | 0.0306 | R_S_oc_sup.transversal | 0.001678 |
| 6 | 0.05 | 0.0390 | R_S_precentral.sup.part | 0.002013 |
| 7 | 0.05 | 0.0468 | L_S_temporal_inf | 0.002349 |
| 8 | 0.05 | 0.0482 | R_S_collat_transv_post | 0.002685 |
| 9 | 0.05 | 0.0540 | R_S_interm_prim.Jensen | 0.003020 |
| 10 | 0.05 | 0.0594 | L_G_front_inf.Triangul | 0.003356 |
| 11 | 0.05 | 0.0639 | L_S_oc_sup.transversal | 0.003691 |
| 12 | 0.05 | 0.0649 | L_G_pariet_inf.Supramar | 0.004027 |
| 13 | 0.05 | 0.0660 | L_S_central | 0.004362 |
| 14 | 0.05 | 0.0681 | L_G_pariet_inf.Angular | 0.004698 |
| 15 | 0.05 | 0.0729 | L_G.S_cingul.Mid.Ant | 0.005034 |
| 16 | 0.05 | 0.0736 | R_G.S_transv_frontopol | 0.005369 |
| 17 | 0.05 | 0.0755 | R_Pole_occipital | 0.005705 |
| 18 | 0.05 | 0.0799 | L_G_front_middle | 0.006040 |
| 19 | 0.05 | 0.0825 | R_S_temporal_inf | 0.006376 |
| 20 | 0.05 | 0.0888 | L_S_precentral.sup.part | 0.006711 |
| 21 | 0.05 | 0.0908 | L_G.S_frontomargin | 0.007047 |
| 22 | 0.05 | 0.0908 | R_S_occipital_ant | 0.007383 |
| 23 | 0.05 | 0.1090 | R_G_temp_sup.Plan_tempo | 0.007718 |
| 24 | 0.05 | 0.1190 | L_S_front_middle | 0.008054 |
| 25 | 0.05 | 0.1190 | R_S_postcentral | 0.008389 |
| 26 | 0.05 | 0.1340 | R_G.S_paracentral | 0.008725 |
| 27 | 0.05 | 0.1530 | R_G_occipital_sup | 0.009060 |
| 28 | 0.05 | 0.1550 | R_G.S_cingul.Mid.Post | 0.009396 |
| 29 | 0.05 | 0.1550 | R_S_intrapariet.P_trans | 0.009732 |
| 30 | 0.05 | 0.1760 | L_S_intrapariet.P_trans | 0.010067 |
| 31 | 0.05 | 0.1810 | L_G_temp_sup.Plan_polar | 0.010403 |
| 32 | 0.05 | 0.1860 | L_S_temporal_sup | 0.010738 |
| 33 | 0.05 | 0.1940 | R_G.S_cingul.Mid.Ant | 0.011074 |
| 34 | 0.05 | 0.1970 | R_G_cuneus | 0.011409 |
| 35 | 0.05 | 0.2060 | R_S_parieto_occipital | 0.011745 |
| 36 | 0.05 | 0.2150 | L_S_calcarine | 0.012081 |
| 37 | 0.05 | 0.2200 | L_G_postcentral | 0.012416 |
| 38 | 0.05 | 0.2280 | R_G.S_subcentral | 0.012752 |
| 39 | 0.05 | 0.2310 | R_G_rectus | 0.013087 |
| 40 | 0.05 | 0.2350 | R_G.S_cingul.Ant | 0.013423 |
| 41 | 0.05 | 0.2370 | L_S_orbital_lateral | 0.013758 |
| 42 | 0.05 | 0.2370 | R_G_front_middle | 0.014094 |
| 43 | 0.05 | 0.2420 | L_S_postcentral | 0.014430 |
| 44 | 0.05 | 0.2530 | L_G_cingul.Post.ventral | 0.014765 |
| 45 | 0.05 | 0.2610 | R_G_precentral | 0.015101 |
| 46 | 0.05 | 0.2620 | L_G.S_paracentral | 0.015436 |
| 47 | 0.05 | 0.2670 | L_S_temporal_transverse | 0.015772 |

| 48 | 0.05 | 0.2690 | mean_thickness | 0.016107 |
| --- | --- | --- | --- | --- |
| 49 | 0.05 | 0.2700 | R_G.S_occipital_inf | 0.016443 |
| 50 | 0.05 | 0.2770 | R_G_parietal_sup | 0.016779 |
| 51 | 0.05 | 0.2860 | R_S_temporal_sup | 0.017114 |
| 52 | 0.05 | 0.2990 | L_G_temp_sup.Lateral | 0.017450 |
| 53 | 0.05 | 0.3040 | R_G_Ins_lg.S_cent_ins | 0.017785 |
| 54 | 0.05 | 0.3050 | L_S_oc.temp_med.Lingual | 0.018121 |
| 55 | 0.05 | 0.3060 | L_G_temp_sup.G_T_transv | 0.018456 |
| 56 | 0.05 | 0.3260 | L_G_cuneus | 0.018792 |
| 57 | 0.05 | 0.3320 | R_G.S_frontomargin | 0.019128 |
| 58 | 0.05 | 0.3330 | R_G_oc.temp_med.Parahip | 0.019463 |
| 59 | 0.05 | 0.3400 | L_G_parietal_sup | 0.019799 |
| 60 | 0.05 | 0.3500 | R_G_temp_sup.Plan_polar | 0.020134 |
| 61 | 0.05 | 0.3510 | L_S_circular_insula_ant | 0.020470 |
| 62 | 0.05 | 0.3930 | L_S_front_sup | 0.020805 |
| 63 | 0.05 | 0.3960 | R_G_pariet_inf.Angular | 0.021141 |
| 64 | 0.05 | 0.4050 | R_S_oc.temp_lat | 0.021477 |
| 65 | 0.05 | 0.4140 | L_S_oc_middle.Lunatus | 0.021812 |
| 66 | 0.05 | 0.4160 | R_G_postcentral | 0.022148 |
| 67 | 0.05 | 0.4170 | L_S_interm_prim.Jensen | 0.022483 |
| 68 | 0.05 | 0.4180 | R_S_temporal_transverse | 0.022819 |
| 69 | 0.05 | 0.4230 | L_S_oc.temp_lat | 0.023154 |
| 70 | 0.05 | 0.4300 | R_Pole_temporal | 0.023490 |
| 71 | 0.05 | 0.4310 | L_S_subparietal | 0.023826 |
| 72 | 0.05 | 0.4510 | L_S_circular_insula_inf | 0.024161 |
| 73 | 0.05 | 0.4570 | L_G_temporal_middle | 0.024497 |
| 74 | 0.05 | 0.4680 | R_S_precentral.inf.part | 0.024832 |
| 75 | 0.05 | 0.4810 | L_Lat_Fis.ant.Vertical | 0.025168 |
| 76 | 0.05 | 0.4840 | R_S_suborbital | 0.025503 |
| 77 | 0.05 | 0.5040 | L_G_oc.temp_med.Parahip | 0.025839 |
| 78 | 0.05 | 0.5060 | L_S_orbital.H_Shaped | 0.026174 |
| 79 | 0.05 | 0.5170 | R_G_oc.temp_lat.fusifor | 0.026510 |
| 80 | 0.05 | 0.5210 | L_S_suborbital | 0.026846 |
| 81 | 0.05 | 0.5270 | L_S_orbital_med.olfact | 0.027181 |
| 82 | 0.05 | 0.5350 | R_G_cingul.Post.dorsal | 0.027517 |
| 83 | 0.05 | 0.5450 | R_S_front_inf | 0.027852 |
| 84 | 0.05 | 0.5480 | L_G_temporal_inf | 0.028188 |
| 85 | 0.05 | 0.5600 | L_G_oc.temp_med.Lingual | 0.028523 |
| 86 | 0.05 | 0.5670 | R_G_front_inf.Triangul | 0.028859 |
| 87 | 0.05 | 0.5710 | L_G.S_occipital_inf | 0.029195 |
| 88 | 0.05 | 0.5720 | R_G_oc.temp_med.Lingual | 0.029530 |
| 89 | 0.05 | 0.5720 | R_G_oc.temp_med.Lingual | 0.029866 |
| 90 | 0.05 | 0.5800 | R_G_pariet_inf.Supramar | 0.030201 |
| 91 | 0.05 | 0.5870 | R_G_front_sup | 0.030537 |
| 92 | 0.05 | 0.5880 | R_S_orbital.H_Shaped | 0.030872 |
| 93 | 0.05 | 0.6000 | L_G.S_cingul.Ant | 0.031208 |
| 94 | 0.05 | 0.6160 | L_Pole_temporal | 0.031544 |
| 95 | 0.05 | 0.6190 | L_G_occipital_middle | 0.031879 |
| 96 | 0.05 | 0.6240 | L_G_precuneus | 0.032215 |
| 97 | 0.05 | 0.6320 | R_G_temporal_inf | 0.032550 |
| 98 | 0.05 | 0.6440 | R_S_calcarine | 0.032886 |

| 99 | 0.05 | 0.6560 | R_G_front_inf.Orbital | 0.033221 |
| --- | --- | --- | --- | --- |
| 100 | 0.05 | 0.6570 | R_G_temporal_middle | 0.033557 |
| 101 | 0.05 | 0.6600 | L_G.S_cingul.Mid.Post | 0.033893 |
| 102 | 0.05 | 0.6610 | L_Pole_occipital | 0.034228 |
| 103 | 0.05 | 0.6690 | L_G_subcallosal | 0.034564 |
| 104 | 0.05 | 0.6720 | L_S_cingul.Marginalis | 0.034899 |
| 105 | 0.05 | 0.6730 | R_S_front_sup | 0.035235 |
| 106 | 0.05 | 0.7170 | R_S_circular_insula_ant | 0.035570 |
| 107 | 0.05 | 0.7320 | R_Lat_Fis.post | 0.035906 |
| 108 | 0.05 | 0.7480 | R_S_cingul.Marginalis | 0.036242 |
| 109 | 0.05 | 0.7530 | R_S_oc.temp_med.Lingual | 0.036577 |
| 110 | 0.05 | 0.7670 | L_Lat_Fis.ant.Horizont | 0.036913 |
| 111 | 0.05 | 0.7760 | L_G_precentral | 0.037248 |
| 112 | 0.05 | 0.7820 | L_G_insular_short | 0.037584 |
| 113 | 0.05 | 0.8120 | L_G_orbital | 0.037919 |
| 114 | 0.05 | 0.8170 | R_S_oc_middle.Lunatus | 0.038255 |
| 115 | 0.05 | 0.8200 | L_G_oc.temp_lat.fusifor | 0.038591 |
| 116 | 0.05 | 0.8200 | L_S_circular_insula_sup | 0.038926 |
| 117 | 0.05 | 0.8210 | R_S_circular_insula_inf | 0.039262 |
| 118 | 0.05 | 0.8250 | L_G_front_inf.Opercular | 0.039597 |
| 119 | 0.05 | 0.8270 | R_S_front_middle | 0.039933 |
| 120 | 0.05 | 0.8310 | R_G_precuneus | 0.040268 |
| 121 | 0.05 | 0.8360 | L_S_parieto_occipital | 0.040604 |
| 122 | 0.05 | 0.8370 | R_Lat_Fis.ant.Horizont | 0.040940 |
| 123 | 0.05 | 0.8460 | R_S_orbital_lateral | 0.041275 |
| 124 | 0.05 | 0.8540 | L_G_front_inf.Orbital | 0.041611 |
| 125 | 0.05 | 0.8540 | L_G_temp_sup.Plan_tempo | 0.041946 |
| 126 | 0.05 | 0.8540 | R_G_temp_sup.Lateral | 0.042282 |
| 127 | 0.05 | 0.8700 | R_Lat_Fis.ant.Vertical | 0.042617 |
| 128 | 0.05 | 0.8710 | R_S_subparietal | 0.042953 |
| 129 | 0.05 | 0.8980 | R_G_insular_short | 0.043289 |
| 130 | 0.05 | 0.9040 | L_G_front_sup | 0.043624 |
| 131 | 0.05 | 0.9090 | L_S_collat_transv_ant | 0.043960 |
| 132 | 0.05 | 0.9100 | L_S_front_inf | 0.044295 |
| 133 | 0.05 | 0.9140 | L_S_occipital_ant | 0.044631 |
| 134 | 0.05 | 0.9190 | R_S_circular_insula_sup | 0.044966 |
| 135 | 0.05 | 0.9250 | L_S_collat_transv_post | 0.045302 |
| 136 | 0.05 | 0.9260 | R_G_front_inf.Opercular | 0.045638 |
| 137 | 0.05 | 0.9320 | L_S_pericallosal | 0.045973 |
| 138 | 0.05 | 0.9330 | R_S_collat_transv_ant | 0.046309 |
| 139 | 0.05 | 0.9330 | R_S_orbital_med.olfact | 0.046644 |
| 140 | 0.05 | 0.9340 | R_S_pericallosal | 0.046980 |
| 141 | 0.05 | 0.9350 | R_G_temp_sup.G_T_transv | 0.047315 |
| 142 | 0.05 | 0.9520 | L_G.S_subcentral | 0.047651 |
| 143 | 0.05 | 0.9640 | L_S_precentral.inf.part | 0.047987 |
| 144 | 0.05 | 0.9700 | L_G_cingul.Post.dorsal | 0.048322 |
| 145 | 0.05 | 0.9700 | L_G_rectus | 0.048658 |
| 146 | 0.05 | 0.9750 | L_G.S_transv_frontopol | 0.048993 |
| 147 | 0.05 | 0.9770 | R_G_cingul.Post.ventral | 0.049329 |
| 148 | 0.05 | 0.9810 | L_G_Ins_lg.S_cent_ins | 0.049664 |
| 149 | 0.05 | 0.9999 | L_Lat_Fis.post | 0.050000 |

**Table S7:** Results from linear mixed effects model predicting remission status from weeks and cortical thickness z-scores

| **Rank** | **Threshold** | **p-value** | **Region** | **Benjamin_Hochberg**  **p-value** | **Odds Ratio** |
| --- | --- | --- | --- | --- | --- |
| 1 | 0.05 | 0.00042 | L_S_central | 0.000336 | 1.423 |
| 2 | 0.05 | 0.00401 | R_S_calcarine | 0.000671 | 1.260 |
| 3 | 0.05 | 0.00457 | L_G.S_cingul.Mid.Ant | 0.001007 | 1.328 |
| 4 | 0.05 | 0.00460 | L_G_pariet_inf.Angular | 0.001342 | 1.399 |
| 5 | 0.05 | 0.00658 | L_G_front_inf.Triangul | 0.001678 | 1.280 |
| 6 | 0.05 | 0.00828 | L_G_postcentral | 0.002013 | 1.257 |
| 7 | 0.05 | 0.00842 | L_G_oc.temp_med.Lingual | 0.002349 | 1.333 |
| 8 | 0.05 | 0.01205 | R_G_postcentral | 0.002685 | 1.262 |
| 9 | 0.05 | 0.01405 | R_S_central | 0.003020 | 1.246 |
| 10 | 0.05 | 0.01456 | R_S_precentral.sup.part | 0.003356 | 1.241 |
| 11 | 0.05 | 0.01580 | L_G_precentral | 0.003691 | 1.253 |
| 12 | 0.05 | 0.01645 | R_G_occipital_middle | 0.004027 | 1.432 |
| 13 | 0.05 | 0.02208 | R_S_parieto_occipital | 0.004362 | 1.362 |
| 14 | 0.05 | 0.02570 | L_G_front_middle | 0.004698 | 1.222 |
| 15 | 0.05 | 0.02582 | R_G_orbital | 0.005034 | 1.299 |
| 16 | 0.05 | 0.03206 | L_S_parieto_occipital | 0.005369 | 1.272 |
| 17 | 0.05 | 0.03250 | L_G_temp_sup.G_T_transv | 0.005705 | 1.207 |
| 18 | 0.05 | 0.03515 | L_G_occipital_sup | 0.006040 | 1.411 |
| 19 | 0.05 | 0.03758 | R_G_precentral | 0.006376 | 1.185 |
| 20 | 0.05 | 0.03975 | L_G.S_paracentral | 0.006711 | 1.200 |
| 21 | 0.05 | 0.04025 | L_S_oc.temp_med.Lingual | 0.007047 | 1.302 |
| 22 | 0.05 | 0.04325 | R_G_temp_sup.Plan_tempo | 0.007383 | 1.211 |
| 23 | 0.05 | 0.04636 | R_G.S_cingul.Ant | 0.007718 | 0.823 |
| 24 | 0.05 | 0.05007 | R_G.S_occipital_inf | 0.008054 | 1.252 |
| 25 | 0.05 | 0.05176 | L_G.S_subcentral | 0.008389 | 1.227 |
| 26 | 0.05 | 0.05277 | R_G_precuneus | 0.008725 | 1.195 |
| 27 | 0.05 | 0.05291 | L_S_oc_middle.Lunatus | 0.009060 | 1.284 |
| 28 | 0.05 | 0.05317 | L_G_parietal_sup | 0.009396 | 1.276 |
| 29 | 0.05 | 0.06062 | L_S_temporal_transverse | 0.009732 | 1.158 |
| 30 | 0.05 | 0.06170 | R_S_oc_sup.transversal | 0.010067 | 1.265 |
| 31 | 0.05 | 0.06216 | R_S_circular_insula_inf | 0.010403 | 0.805 |
| 32 | 0.05 | 0.06695 | R_G_oc.temp_med.Lingual | 0.010738 | 1.168 |
| 33 | 0.05 | 0.06762 | L_S_precentral.sup.part | 0.011074 | 1.237 |
| 34 | 0.05 | 0.07218 | L_G_cingul.Post.ventral | 0.011409 | 1.089 |
| 35 | 0.05 | 0.07334 | L_S_precentral.inf.part | 0.011745 | 1.111 |
| 36 | 0.05 | 0.07430 | L_S_temporal_inf | 0.012081 | 1.324 |
| 37 | 0.05 | 0.07622 | R_S_front_inf | 0.012416 | 0.845 |
| 38 | 0.05 | 0.07788 | L_G_front_inf.Opercular | 0.012752 | 1.112 |
| 39 | 0.05 | 0.08217 | R_G_front_middle | 0.013087 | 1.095 |
| 40 | 0.05 | 0.08513 | L_S_postcentral | 0.013423 | 1.277 |
| 41 | 0.05 | 0.08797 | mean_thickness | 0.013758 | 1.240 |
| 42 | 0.05 | 0.08805 | R_G_pariet_inf.Angular | 0.014094 | 1.211 |
| 43 | 0.05 | 0.09216 | L_G_cuneus | 0.014430 | 1.180 |
| 44 | 0.05 | 0.09420 | R_G_pariet_inf.Supramar | 0.014765 | 1.150 |
| 45 | 0.05 | 0.09784 | L_S_temporal_sup | 0.015101 | 1.195 |

| 46 | 0.05 | 0.10965 | L_G.S_frontomargin | 0.015436 | 1.031 |
| --- | --- | --- | --- | --- | --- |
| 47 | 0.05 | 0.11495 | R_S_precentral.inf.part | 0.015772 | 1.137 |
| 48 | 0.05 | 0.11550 | L_S_intrapariet.P_trans | 0.016107 | 1.243 |
| 49 | 0.05 | 0.12473 | R_S_interm_prim.Jensen | 0.016443 | 1.127 |
| 50 | 0.05 | 0.12480 | L_S_front_sup | 0.016779 | 1.163 |
| 51 | 0.05 | 0.13178 | R_S_postcentral | 0.017114 | 1.214 |
| 52 | 0.05 | 0.13518 | L_S_orbital_lateral | 0.017450 | 1.104 |
| 53 | 0.05 | 0.16935 | L_G_temp_sup.Plan_tempo | 0.017785 | 1.205 |
| 54 | 0.05 | 0.16968 | R_G.S_cingul.Mid.Post | 0.018121 | 1.146 |
| 55 | 0.05 | 0.17033 | L_Lat_Fis.post | 0.018456 | 1.221 |
| 56 | 0.05 | 0.17195 | L_G_insular_short | 0.018792 | 0.999 |
| 57 | 0.05 | 0.17392 | R_G_occipital_sup | 0.019128 | 1.233 |
| 58 | 0.05 | 0.17930 | R_S_intrapariet.P_trans | 0.019463 | 1.180 |
| 59 | 0.05 | 0.19368 | R_G_Ins_lg.S_cent_ins | 0.019799 | 1.165 |
| 60 | 0.05 | 0.19682 | R_G_front_inf.Triangul | 0.020134 | 1.096 |
| 61 | 0.05 | 0.19739 | R_S_circular_insula_sup | 0.020470 | 0.980 |
| 62 | 0.05 | 0.19862 | R_S_occipital_ant | 0.020805 | 1.243 |
| 63 | 0.05 | 0.19870 | R_G.S_subcentral | 0.021141 | 1.043 |
| 64 | 0.05 | 0.21421 | L_S_front_middle | 0.021477 | 1.145 |
| 65 | 0.05 | 0.21592 | L_G_pariet_inf.Suprama | 0.021812 | 1.205 |
| 66 | 0.05 | 0.21593 | L_G_oc.temp_med.Parahip | 0.022148 | 1.088 |
| 67 | 0.05 | 0.21713 | R_G.S_paracentral | 0.022483 | 1.139 |
| 68 | 0.05 | 0.22799 | L_G_temporal_middle | 0.022819 | 1.175 |
| 69 | 0.05 | 0.23427 | L_S_oc_sup.transversal | 0.023154 | 1.249 |
| 70 | 0.05 | 0.24050 | R_G_cuneus | 0.023490 | 1.250 |
| 71 | 0.05 | 0.24319 | L_G.S_cingul.Ant | 0.023826 | 1.047 |
| 72 | 0.05 | 0.24333 | R_S_temporal_inf | 0.024161 | 1.188 |
| 73 | 0.05 | 0.24583 | R_G_temp_sup.G_T_transv | 0.024497 | 1.034 |
| 74 | 0.05 | 0.25917 | R_G_temporal_inf | 0.024832 | 1.174 |
| 75 | 0.05 | 0.26954 | R_G_cingul.Post.ventral | 0.025168 | 1.084 |
| 76 | 0.05 | 0.29480 | R_G_parietal_sup | 0.025503 | 1.229 |
| 77 | 0.05 | 0.29818 | L_S_calcarine | 0.025839 | 1.098 |
| 78 | 0.05 | 0.29826 | R_S_temporal_sup | 0.026174 | 1.154 |
| 79 | 0.05 | 0.30826 | R_G.S_frontomargin | 0.026510 | 0.978 |
| 80 | 0.05 | 0.31199 | L_Lat_Fis.ant.Horizont | 0.026846 | 1.038 |
| 81 | 0.05 | 0.33101 | R_G_temporal_middle | 0.027181 | 1.137 |
| 82 | 0.05 | 0.33408 | L_S_cingul.Marginalis | 0.027517 | 1.194 |
| 83 | 0.05 | 0.33686 | L_G_front_inf.Orbital | 0.027852 | 0.959 |
| 84 | 0.05 | 0.33819 | R_S_orbital.H_Shaped | 0.028188 | 0.989 |
| 85 | 0.05 | 0.34613 | L_S_collat_transv_post | 0.028523 | 1.225 |
| 86 | 0.05 | 0.35991 | L_S_circular_insula_inf | 0.028859 | 1.071 |
| 87 | 0.05 | 0.36393 | R_G_temp_sup.Lateral | 0.029195 | 1.034 |
| 88 | 0.05 | 0.38261 | L_G_occipital_middle | 0.029530 | 1.164 |
| 89 | 0.05 | 0.39391 | R_G_insular_short | 0.029866 | 1.009 |
| 90 | 0.05 | 0.39678 | L_S_front_inf | 0.030201 | 1.086 |
| 91 | 0.05 | 0.41204 | R_Lat_Fis.post | 0.030537 | 1.134 |
| 92 | 0.05 | 0.42543 | R_G_temp_sup.Plan_polar | 0.030872 | 1.128 |
| 93 | 0.05 | 0.42884 | L_G.S_occipital_inf | 0.031208 | 1.240 |
| 94 | 0.05 | 0.42956 | R_S_front_sup | 0.031544 | 1.009 |
| 95 | 0.05 | 0.44390 | L_Pole_occipital | 0.031879 | 1.115 |
| 96 | 0.05 | 0.45370 | R_S_temporal_transverse | 0.032215 | 1.103 |

| 97 | 0.05 | 0.46489 | L_S_collat_transv_ant | 0.032550 | 1.161 |
| --- | --- | --- | --- | --- | --- |
| 98 | 0.05 | 0.48128 | R_G_oc.temp_med.Parahip | 0.032886 | 1.041 |
| 99 | 0.05 | 0.48510 | R_G_temp_sup.G_T_transv | 0.033221 | 1.034 |
| 100 | 0.05 | 0.48979 | L_Lat_Fis.ant.Vertical | 0.033557 | 1.001 |
| 101 | 0.05 | 0.49192 | L_G_Ins_lg.S_cent_ins | 0.033893 | 1.006 |
| 102 | 0.05 | 0.49657 | R_G_rectus | 0.034228 | 1.042 |
| 103 | 0.05 | 0.50148 | R_S_orbital_lateral | 0.034564 | 0.905 |
| 104 | 0.05 | 0.53661 | R_G_subcallosal | 0.034899 | 1.177 |
| 105 | 0.05 | 0.54216 | R_G_oc.temp_lat.fusifor | 0.035235 | 1.046 |
| 106 | 0.05 | 0.56476 | L_S_interm_prim.Jensen | 0.035570 | 0.990 |
| 107 | 0.05 | 0.57168 | L_G_orbital | 0.035906 | 1.030 |
| 108 | 0.05 | 0.57520 | L_S_pericallosal | 0.036242 | 1.005 |
| 109 | 0.05 | 0.57534 | L_G_front_sup | 0.036577 | 1.043 |
| 110 | 0.05 | 0.59451 | L_S_orbital_med.olfact | 0.036913 | 1.21 |
| 111 | 0.05 | 0.60591 | R_G_front_inf.Opercular | 0.037248 | 0.972 |
| 112 | 0.05 | 0.61548 | R_S_orbital_med.olfact | 0.037584 | 0.905 |
| 113 | 0.05 | 0.61647 | L_G_precuneus | 0.037919 | 1.107 |
| 114 | 0.05 | 0.64192 | R_G.S_transv_frontopol | 0.038255 | 1.000 |
| 115 | 0.05 | 0.69730 | R_S_collat_transv_ant | 0.038591 | 0.987 |
| 116 | 0.05 | 0.69908 | R_Pole_temporal | 0.038926 | 0.957 |
| 117 | 0.05 | 0.70625 | L_Pole_temporal | 0.039262 | 1.055 |
| 118 | 0.05 | 0.71851 | L_G_subcallosal | 0.039597 | 0.998 |
| 119 | 0.05 | 0.73934 | R_G.S_cingul.Mid.Ant | 0.039933 | 0.896 |
| 120 | 0.05 | 0.74158 | L_S_oc.temp_lat | 0.040268 | 1.020 |
| 121 | 0.05 | 0.74824 | L_G_oc.temp_lat.fusifor | 0.040604 | 1.080 |
| 122 | 0.05 | 0.75468 | R_S_oc.temp_med.Lingual | 0.040940 | 1.014 |
| 123 | 0.05 | 0.76742 | R_S_subparietal | 0.041275 | 1.100 |
| 124 | 0.05 | 0.77471 | L_G.S_cingul.Mid.Post | 0.041611 | 1.010 |
| 125 | 0.05 | 0.78146 | R_G_cingul.Post.dorsal | 0.041946 | 1.039 |
| 126 | 0.05 | 0.79092 | R_S_oc.temp_lat | 0.042282 | 1.074 |
| 127 | 0.05 | 0.79901 | R_S_suborbital | 0.042617 | 0.962 |
| 128 | 0.05 | 0.79955 | L_G_temp_sup.Lateral | 0.042953 | 0.974 |
| 129 | 0.05 | 0.80265 | R_S_pericallosal | 0.043289 | 1.038 |
| 130 | 0.05 | 0.81617 | R_S_circular_insula_ant | 0.043624 | 0.963 |
| 131 | 0.05 | 0.82262 | L_G_temporal_inf | 0.043960 | 1.075 |
| 132 | 0.05 | 0.82875 | R_G_front_inf.Orbital | 0.044295 | 0.921 |
| 133 | 0.05 | 0.82918 | L_S_subparietal | 0.044631 | 1.047 |
| 134 | 0.05 | 0.83460 | R_S_collat_transv_post | 0.044966 | 0.979 |
| 135 | 0.05 | 0.83567 | L_S_suborbital | 0.045302 | 0.958 |
| 136 | 0.05 | 0.83818 | L_S_orbital.H_Shaped | 0.045638 | 1.050 |
| 137 | 0.05 | 0.85663 | R_G_front_sup | 0.045973 | 0.976 |
| 138 | 0.05 | 0.88765 | R_S_cingul.Marginalis | 0.046309 | 1.038 |
| 139 | 0.05 | 0.89381 | L_G_rectus | 0.046644 | 1.018 |
| 140 | 0.05 | 0.89389 | L_S_circular_insula_ant | 0.046980 | 0.992 |
| 141 | 0.05 | 0.90962 | R_Lat_Fis.ant.Horizont | 0.047315 | 0.872 |
| 142 | 0.05 | 0.93098 | R_S_oc_middle.Lunatus | 0.047651 | 1.068 |
| 143 | 0.05 | 0.94295 | L_S_circular_insula_sup | 0.047987 | 1.075 |
| 144 | 0.05 | 0.95414 | L_G_temp_sup.Plan_polar | 0.048322 | 0.945 |
| 145 | 0.05 | 0.96490 | L_G.S_transv_frontopol | 0.048658 | 1.007 |
| 146 | 0.05 | 0.98590 | L_G_cingul.Post.dorsal | 0.048993 | 0.980 |
| 147 | 0.05 | 0.99349 | R_Pole_occipital | 0.049329 | 0.995 |

| 148 | 0.05 | 0.99450 | R_S_front_middle | 0.049664 | 0.959 |
| --- | --- | --- | --- | --- | --- |
| 149 | 0.05 | 0.99759 | L_S_occipital_ant | 0.050000 | 1.039 |

**Table S8:** Results from linear mixed effects model predicting the sum of the PANSS items used to define remission over the medium-term symptoms from weeks and cortical thickness z-scores

| **Rank** | **Threshold** | **p-value** | **Region** | **Benjamin_Hochberg p-value** |
| --- | --- | --- | --- | --- |
| 1 | 0.05 | 0.003 | L_S_temporal_inf | 0.000336 |
| 2 | 0.05 | 0.004 | L_G_occipital_sup | 0.000671 |
| 3 | 0.05 | 0.008 | R_G_temp_sup.Plan_tempo | 0.001007 |
| 4 | 0.05 | 0.009 | R_S_temporal_transverse | 0.001342 |
| 5 | 0.05 | 0.010 | R_G_orbital | 0.001678 |
| 6 | 0.05 | 0.013 | L_G_pariet_inf.Supramar | 0.002013 |
| 7 | 0.05 | 0.013 | R_S_precentral.sup.part | 0.002349 |
| 8 | 0.05 | 0.014 | R_G_occipital_middle | 0.002685 |
| 9 | 0.05 | 0.015 | L_G_temporal_middle | 0.003020 |
| 10 | 0.05 | 0.018 | R_G_Ins_lg.S_cent_ins | 0.003356 |
| 11 | 0.05 | 0.023 | L_G_postcentral | 0.003691 |
| 12 | 0.05 | 0.025 | L_G_front_middle | 0.004027 |
| 13 | 0.05 | 0.032 | L_S_intrapariet.P_trans | 0.004362 |
| 14 | 0.05 | 0.033 | L_S_central | 0.004698 |
| 15 | 0.05 | 0.033 | R_G.S_occipital_inf | 0.005034 |
| 16 | 0.05 | 0.034 | L_G_pariet_inf.Angular | 0.005369 |
| 17 | 0.05 | 0.045 | L_S_postcentral | 0.005705 |
| 18 | 0.05 | 0.052 | L_G_temp_sup.Lateral | 0.006040 |
| 19 | 0.05 | 0.055 | R_G.S_paracentral | 0.006376 |
| 20 | 0.05 | 0.059 | mean_thickness | 0.006711 |
| 21 | 0.05 | 0.065 | L_S_front_middle | 0.007047 |
| 22 | 0.05 | 0.066 | R_S_central | 0.007383 |
| 23 | 0.05 | 0.069 | L_S_oc.temp_med.Lingual | 0.007718 |
| 24 | 0.05 | 0.073 | R_S_oc_sup.transversal | 0.008054 |
| 25 | 0.05 | 0.075 | L_S_temporal_sup | 0.008389 |
| 26 | 0.05 | 0.078 | L_G.S_subcentral | 0.008725 |
| 27 | 0.05 | 0.082 | R_S_temporal_inf | 0.009060 |
| 28 | 0.05 | 0.083 | R_S_occipital_ant | 0.009396 |
| 29 | 0.05 | 0.088 | R_S_parieto_occipital | 0.009732 |
| 30 | 0.05 | 0.091 | R_S_front_inf | 0.010067 |
| 31 | 0.05 | 0.105 | L_S_oc_sup.transversal | 0.010403 |
| 32 | 0.05 | 0.108 | L_G_front_inf.Opercular | 0.010738 |
| 33 | 0.05 | 0.113 | R_G.S_cingul.Mid.Post | 0.011074 |
| 34 | 0.05 | 0.117 | R_S_postcentral | 0.011409 |
| 35 | 0.05 | 0.119 | R_Lat_Fis.ant.Horizont | 0.011745 |
| 36 | 0.05 | 0.122 | R_S_oc.temp_lat | 0.012081 |
| 37 | 0.05 | 0.124 | R_G_postcentral | 0.012416 |
| 38 | 0.05 | 0.127 | L_G.S_paracentral | 0.012752 |
| 39 | 0.05 | 0.131 | L_G_temp_sup.Plan_tempo | 0.013087 |
| 40 | 0.05 | 0.132 | L_S_precentral.sup.part | 0.013423 |
| 41 | 0.05 | 0.143 | L_G_temp_sup.Plan_polar | 0.013758 |
| 42 | 0.05 | 0.150 | L_G_Ins_lg.S_cent_ins | 0.014094 |
| 43 | 0.05 | 0.150 | R_Lat_Fis.ant.Vertical | 0.014430 |
| 44 | 0.05 | 0.153 | R_G_precentral | 0.014765 |
| 45 | 0.05 | 0.155 | R_S_intrapariet.P_trans | 0.015101 |
| 46 | 0.05 | 0.157 | R_S_interm_prim.Jensen | 0.015436 |

| 47 | 0.05 | 0.165 | L_G_orbital | 0.015772 |
| --- | --- | --- | --- | --- |
| 48 | 0.05 | 0.166 | R_G_temp_sup.Plan_polar | 0.016107 |
| 49 | 0.05 | 0.167 | R_G.S_cingul.Mid.Ant | 0.016443 |
| 50 | 0.05 | 0.168 | L_S_oc_middle.Lunatus | 0.016779 |
| 51 | 0.05 | 0.172 | L_S_front_sup | 0.017114 |
| 52 | 0.05 | 0.182 | L_G_parietal_sup | 0.017450 |
| 53 | 0.05 | 0.184 | R_G_oc.temp_med.Lingual | 0.017785 |
| 54 | 0.05 | 0.185 | R_S_temporal_sup | 0.018121 |
| 55 | 0.05 | 0.187 | L_S_orbital_lateral | 0.018456 |
| 56 | 0.05 | 0.189 | R_G_cuneus | 0.018792 |
| 57 | 0.05 | 0.192 | L_S_circular_insula_inf | 0.019128 |
| 58 | 0.05 | 0.197 | R_G_parietal_sup | 0.019463 |
| 59 | 0.05 | 0.197 | R_G_temporal_inf | 0.019799 |
| 60 | 0.05 | 0.199 | R_S_collat_transv_post | 0.020134 |
| 61 | 0.05 | 0.206 | L_S_collat_transv_post | 0.020470 |
| 62 | 0.05 | 0.209 | L_G_temp_sup.G_T_transv | 0.020805 |
| 63 | 0.05 | 0.219 | R_G.S_cingul.Ant | 0.021141 |
| 64 | 0.05 | 0.244 | R_G_front_middle | 0.021477 |
| 65 | 0.05 | 0.248 | R_G_pariet_inf.Angular | 0.021812 |
| 66 | 0.05 | 0.249 | R_S_subparietal | 0.022148 |
| 67 | 0.05 | 0.261 | R_Lat_Fis.post | 0.022483 |
| 68 | 0.05 | 0.264 | L_G_oc.temp_med.Lingual | 0.022819 |
| 69 | 0.05 | 0.273 | L_G_cuneus | 0.023154 |
| 70 | 0.05 | 0.275 | R_G_temporal_middle | 0.023490 |
| 71 | 0.05 | 0.297 | R_G_oc.temp_lat.fusifor | 0.023826 |
| 72 | 0.05 | 0.308 | R_G_cingul.Post.dorsal | 0.024161 |
| 73 | 0.05 | 0.315 | L_Lat_Fis.post | 0.024497 |
| 74 | 0.05 | 0.315 | R_G_occipital_sup | 0.024832 |
| 75 | 0.05 | 0.316 | R_G.S_frontomargin | 0.025168 |
| 76 | 0.05 | 0.318 | L_S_orbital.H_Shaped | 0.025503 |
| 77 | 0.05 | 0.320 | R_G_temp_sup.G_T_transv | 0.025839 |
| 78 | 0.05 | 0.331 | L_S_parieto_occipital | 0.026174 |
| 79 | 0.05 | 0.332 | R_G_subcallosal | 0.026510 |
| 80 | 0.05 | 0.339 | R_G_precuneus | 0.026846 |
| 81 | 0.05 | 0.345 | R_G_rectus | 0.027181 |
| 82 | 0.05 | 0.350 | R_G_cingul.Post.ventral | 0.027517 |
| 83 | 0.05 | 0.351 | L_S_temporal_transverse | 0.027852 |
| 84 | 0.05 | 0.383 | L_G_oc.temp_med.Parahip | 0.028188 |
| 85 | 0.05 | 0.394 | L_G_precentral | 0.028523 |
| 86 | 0.05 | 0.402 | L_Pole_temporal | 0.028859 |
| 87 | 0.05 | 0.427 | R_S_collat_transv_ant | 0.029195 |
| 88 | 0.05 | 0.434 | L_G.S_cingul.Mid.Ant | 0.029530 |
| 89 | 0.05 | 0.438 | R_S_pericallosal | 0.029866 |
| 90 | 0.05 | 0.452 | R_G_front_inf.Opercular | 0.030201 |
| 91 | 0.05 | 0.457 | L_G_front_inf.Triangul | 0.030537 |
| 92 | 0.05 | 0.458 | R_G_pariet_inf.Supramar | 0.030872 |
| 93 | 0.05 | 0.460 | R_Pole_temporal | 0.031208 |
| 94 | 0.05 | 0.465 | R_S_precentral.inf.part | 0.031544 |
| 95 | 0.05 | 0.483 | R_S_calcarine | 0.031879 |
| 96 | 0.05 | 0.489 | L_G.S_frontomargin | 0.032215 |
| 97 | 0.05 | 0.499 | L_G_temporal_inf | 0.032550 |

| 98 | 0.05 | 0.500 | R_Pole_occipital | 0.032886 |
| --- | --- | --- | --- | --- |
| 99 | 0.05 | 0.507 | R_G_insular_short | 0.033221 |
| 100 | 0.05 | 0.518 | R_S_orbital_lateral | 0.033557 |
| 101 | 0.05 | 0.523 | R_G_front_sup | 0.033893 |
| 102 | 0.05 | 0.538 | L_S_occipital_ant | 0.034228 |
| 103 | 0.05 | 0.549 | R_G_temp_sup.Lateral | 0.034564 |
| 104 | 0.05 | 0.558 | L_G.S_occipital_inf | 0.034899 |
| 105 | 0.05 | 0.577 | L_S_calcarine | 0.035235 |
| 106 | 0.05 | 0.611 | L_S_collat_transv_ant | 0.035570 |
| 107 | 0.05 | 0.611 | L_S_orbital_med.olfact | 0.035906 |
| 108 | 0.05 | 0.621 | R_S_orbital_med.olfact | 0.036242 |
| 109 | 0.05 | 0.632 | L_S_front_inf | 0.036577 |
| 110 | 0.05 | 0.646 | L_S_precentral.inf.part | 0.036913 |
| 111 | 0.05 | 0.647 | L_G_cingul.Post.ventral | 0.037248 |
| 112 | 0.05 | 0.650 | R_G.S_transv_frontopol | 0.037584 |
| 113 | 0.05 | 0.657 | L_G_oc.temp_lat.fusifor | 0.037919 |
| 114 | 0.05 | 0.659 | R_S_circular_insula_inf | 0.038255 |
| 115 | 0.05 | 0.661 | L_S_cingul.Marginalis | 0.038591 |
| 116 | 0.05 | 0.663 | L_G_insular_short | 0.038926 |
| 117 | 0.05 | 0.665 | R_S_suborbital | 0.039262 |
| 118 | 0.05 | 0.667 | L_G_front_sup | 0.039597 |
| 119 | 0.05 | 0.691 | L_S_subparietal | 0.039933 |
| 120 | 0.05 | 0.702 | L_G_precuneus | 0.040268 |
| 121 | 0.05 | 0.708 | R_G_oc.temp_med.Parahip | 0.040604 |
| 122 | 0.05 | 0.711 | L_G.S_cingul.Ant | 0.040940 |
| 123 | 0.05 | 0.714 | R_S_front_sup | 0.041275 |
| 124 | 0.05 | 0.757 | L_S_suborbital | 0.041611 |
| 125 | 0.05 | 0.766 | L_S_oc.temp_lat | 0.041946 |
| 126 | 0.05 | 0.766 | R_S_oc.temp_med.Lingual | 0.042282 |
| 127 | 0.05 | 0.773 | L_G_subcallosal | 0.042617 |
| 128 | 0.05 | 0.779 | R_S_orbital.H_Shaped | 0.042953 |
| 129 | 0.05 | 0.781 | L_S_circular_insula_sup | 0.043289 |
| 130 | 0.05 | 0.782 | L_Pole_occipital | 0.043624 |
| 131 | 0.05 | 0.794 | L_G.S_transv_frontopol | 0.043960 |
| 132 | 0.05 | 0.828 | L_Lat_Fis.ant.Horizont | 0.044295 |
| 133 | 0.05 | 0.836 | L_G_rectus | 0.044631 |
| 134 | 0.05 | 0.842 | L_G.S_cingul.Mid.Post | 0.044966 |
| 135 | 0.05 | 0.842 | R_G.S_subcentral | 0.045302 |
| 136 | 0.05 | 0.848 | R_S_cingul.Marginalis | 0.045638 |
| 137 | 0.05 | 0.857 | L_G_occipital_middle | 0.045973 |
| 138 | 0.05 | 0.864 | L_S_pericallosal | 0.046309 |
| 139 | 0.05 | 0.872 | L_S_circular_insula_ant | 0.046644 |
| 140 | 0.05 | 0.880 | L_G_cingul.Post.dorsal | 0.046980 |
| 141 | 0.05 | 0.888 | L_G_front_inf.Orbital | 0.047315 |
| 142 | 0.05 | 0.894 | R_S_front_middle | 0.047651 |
| 143 | 0.05 | 0.913 | R_S_oc_middle.Lunatus | 0.047987 |
| 144 | 0.05 | 0.938 | L_Lat_Fis.ant.Vertical | 0.048322 |
| 145 | 0.05 | 0.947 | L_S_interm_prim.Jensen | 0.048658 |
| 146 | 0.05 | 0.978 | R_S_circular_insula_ant | 0.048993 |
| 147 | 0.05 | 0.982 | R_S_circular_insula_sup | 0.049329 |
| 148 | 0.05 | 0.991 | R_G_front_inf.Orbital | 0.049664 |

149 0.05 0.996 R_G_front_inf.Triangul 0.050000

**Table S9:** Results from regional Mann-Witney U tests comparing distributions of z-score between FEP patients and healthy participants after exclusion of smaller sites.

| **Rank** | **Threshold** | **p-value** | **Region** | **Benjamin_Hochberg p-value** |
| --- | --- | --- | --- | --- |
| 1 | 0.05 | 0.000057 | L_G_front_middle | 0.000338 |
| 2 | 0.05 | 0.000057 | R_G_front_sup | 0.000676 |
| 3 | 0.05 | 0.000120 | L_G_precuneus | 0.001014 |
| 4 | 0.05 | 0.000264 | L_GS_frontomarg | 0.001351 |
| 5 | 0.05 | 0.000422 | L_G_pariet_infAngular | 0.001689 |
| 6 | 0.05 | 0.000544 | R_GS_frontomargin | 0.002027 |
| 7 | 0.05 | 0.000594 | L_S_cingulMarginalis | 0.002365 |
| 8 | 0.05 | 0.000642 | L_G_octemp_medLingual | 0.002703 |
| 9 | 0.05 | 0.000765 | L_S_precentralinfpart | 0.003040 |
| 10 | 0.05 | 0.000807 | R_G_temp_supLateral | 0.003378 |
| 11 | 0.05 | 0.000854 | R_G_precuneus | 0.003716 |
| 12 | 0.05 | 0.001223 | L_GS_subcentral | 0.004054 |
| 13 | 0.05 | 0.001406 | L_G_octemp_latfusifor | 0.004392 |
| 14 | 0.05 | 0.001815 | L_GS_occipital_inf | 0.004730 |
| 15 | 0.05 | 0.001882 | L_S_parieto_occipital | 0.005068 |
| 16 | 0.05 | 0.002405 | R_GS_cingulMidAnt | 0.005405 |
| 17 | 0.05 | 0.001673 | R_G_occipital_sup | 0.005743 |
| 18 | 0.05 | 0.002163 | R_S_cingulMarginalis | 0.006081 |
| 19 | 0.05 | 0.002756 | L_G_front_sup | 0.006419 |
| 20 | 0.05 | 0.003032 | L_S_intraparietP_trans | 0.006757 |
| 21 | 0.05 | 0.002967 | R_G_front_infOpercular | 0.007095 |
| 22 | 0.05 | 0.004105 | L_S_front_inf | 0.007432 |
| 23 | 0.05 | 0.003600 | R_G_cuneus | 0.007770 |
| 24 | 0.05 | 0.003853 | R_Pole_occipital | 0.008108 |
| 25 | 0.05 | 0.003616 | R_S_octemp_medLingual | 0.008446 |
| 26 | 0.05 | 0.003631 | R_S_orbitalH_Shaped | 0.008784 |
| 27 | 0.05 | 0.003789 | R_S_temporal_inf | 0.009122 |
| 28 | 0.05 | 0.004953 | L_S_circular_insula_inf | 0.009459 |
| 29 | 0.05 | 0.004655 | R_G_front_middle | 0.009797 |
| 30 | 0.05 | 0.005790 | L_S_temporal_inf | 0.010135 |
| 31 | 0.05 | 0.006407 | R_G_parietal_sup | 0.010473 |
| 32 | 0.05 | 0.006254 | R_G_temp_supPlan_tempo | 0.010810 |
| 33 | 0.05 | 0.006279 | R_S_circular_insula_sup | 0.011149 |
| 34 | 0.05 | 0.006485 | R_S_precentralsuppart | 0.011486 |
| 35 | 0.05 | 0.006860 | L_G_front_infOpercular | 0.011824 |
| 36 | 0.05 | 0.007460 | R_G_occipital_middle | 0.012162 |
| 37 | 0.05 | 0.006971 | R_S_parieto_occipital | 0.012500 |
| 38 | 0.05 | 0.008074 | R_G_rectus | 0.012838 |
| 39 | 0.05 | 0.008042 | R_S_temporal_sup | 0.013176 |
| 40 | 0.05 | 0.008870 | L_GS_transv_frontopol | 0.013514 |
| 41 | 0.05 | 0.008597 | L_S_precentralsuppart | 0.013851 |
| 42 | 0.05 | 0.010235 | R_G_octemp_medLingual | 0.014189 |
| 43 | 0.05 | 0.009549 | L_G_pariet_sup | 0.014527 |
| 44 | 0.05 | 0.010554 | R_S_collat_transv_post | 0.014865 |
| 45 | 0.05 | 0.010799 | L_S_front_sup | 0.015203 |
| 46 | 0.05 | 0.011007 | L_G_front_infTriangul | 0.015541 |
| 47 | 0.05 | 0.012055 | L_G_temp_supG_T_transv | 0.015878 |

| 48 | 0.05 | 0.011964 | R_GS_cingulMidAnt | 0.016216 |
| --- | --- | --- | --- | --- |
| 49 | 0.05 | 0.012946 | R_S_subparietal | 0.016554 |
| 50 | 0.05 | 0.012659 | L_S_front_middle | 0.016892 |
| 51 | 0.05 | 0.014051 | L_S_orbitalH_Shaped | 0.017230 |
| 52 | 0.05 | 0.014473 | R_S_precentralinfpart | 0.017568 |
| 53 | 0.05 | 0.015126 | R_G_pariet_infAngular | 0.017905 |
| 54 | 0.05 | 0.015922 | R_S_front_sup | 0.018243 |
| 55 | 0.05 | 0.015806 | L_S_circular_insula_sup | 0.018581 |
| 56 | 0.05 | 0.016937 | L_G_occipital_middle | 0.018919 |
| 57 | 0.05 | 0.017497 | R_GS_transv_frontopol | 0.019257 |
| 58 | 0.05 | 0.017560 | L_S_oc_middleLunatus | 0.019595 |
| 59 | 0.05 | 0.017944 | L_S_postcentral | 0.019932 |
| 60 | 0.05 | 0.017624 | R_G_temp_supG_T_transv | 0.020270 |
| 61 | 0.05 | 0.018533 | L_S_suborbital | 0.020608 |
| 62 | 0.05 | 0.020403 | L_G_temp_supPlan_tempo | 0.020946 |
| 63 | 0.05 | 0.021663 | L_S_octemp_medLingual | 0.021284 |
| 64 | 0.05 | 0.024136 | R_Lat_Fispost | 0.021622 |
| 65 | 0.05 | 0.024642 | R_G_temporal_middle | 0.021960 |
| 66 | 0.05 | 0.025681 | R_S_intraparietP_trans | 0.022297 |
| 67 | 0.05 | 0.028351 | L_GS_cingulMidPost | 0.022635 |
| 68 | 0.05 | 0.028255 | R_S_octemp_lat | 0.022973 |
| 69 | 0.05 | 0.031677 | R_Lat_FisantVertical | 0.023311 |
| 70 | 0.05 | 0.032640 | L_G_cuneus | 0.023649 |
| 71 | 0.05 | 0.034301 | R_S_front_inf | 0.023987 |
| 72 | 0.05 | 0.036987 | R_S_front_middle | 0.024324 |
| 73 | 0.05 | 0.042357 | R_G_subcallosal | 0.024662 |
| 74 | 0.05 | 0.043313 | L_S_subparietal | 0.025000 |
| 75 | 0.05 | 0.043868 | L_GS_cingulAnt | 0.025339 |
| 76 | 0.05 | 0.043729 | L_G_orbital | 0.025676 |
| 77 | 0.05 | 0.045137 | R_G_partiet_infSupramar | 0.026014 |
| 78 | 0.05 | 0.046292 | R_GS_paracentral | 0.026351 |
| 79 | 0.05 | 0.048828 | L_G_temp_supLateral | 0.026689 |
| 80 | 0.05 | 0.049134 | L_G_temporal_inf | 0.027027 |
| 81 | 0.05 | 0.052605 | R_GS_occipital_inf | 0.027365 |
| 82 | 0.05 | 0.056109 | L_S_temporal_transverse | 0.027703 |
| 83 | 0.05 | 0.056624 | R_G_octemp_latfusifor | 0.028041 |
| 84 | 0.05 | 0.061448 | L_G_occipital_sup | 0.028378 |
| 85 | 0.05 | 0.062941 | L_G_pariet_infSupramar | 0.028716 |
| 86 | 0.05 | 0.066213 | L_Pole_occipital | 0.029054 |
| 87 | 0.05 | 0.067202 | L_S_orbital_lateral | 0.029392 |
| 88 | 0.05 | 0.067801 | L_Lat_Fispost | 0.029729 |
| 89 | 0.05 | 0.068809 | R_S_postcentral | 0.030068 |
| 90 | 0.05 | 0.073178 | L_S_temporal_sup | 0.030405 |
| 91 | 0.05 | 0.072965 | L_G_precentral | 0.030743 |
| 92 | 0.05 | 0.079811 | R_S_temporal_transverse | 0.031081 |
| 93 | 0.05 | 0.081427 | L_S_oc_suptransversal | 0.031419 |
| 94 | 0.05 | 0.082362 | L_G_postcentral | 0.031757 |
| 95 | 0.05 | 0.082597 | R_S_oc_suptransversal | 0.032095 |
| 96 | 0.05 | 0.093235 | L_G_cingulPostdorsal | 0.032432 |
| 97 | 0.05 | 0.096393 | L_S_occipital_ant | 0.032770 |
| 98 | 0.05 | 0.097195 | L_G_front_infOrbital | 0.033108 |

| 99 | 0.05 | 0.106380 | L_G_temporal_middle | 0.033446 |
| --- | --- | --- | --- | --- |
| 100 | 0.05 | 0.117483 | L_GS_paracentral | 0.033784 |
| 101 | 0.05 | 0.117795 | R_GS_cingulMidPost | 0.034121 |
| 102 | 0.05 | 0.122558 | L_S_interm_primJensen | 0.034460 |
| 103 | 0.05 | 0.130827 | R_G_cingulPostdorsal | 0.034797 |
| 104 | 0.05 | 0.141314 | R_G_temporal_inf | 0.035135 |
| 105 | 0.05 | 0.142036 | L_S_collat_transv_post | 0.035473 |
| 106 | 0.05 | 0.142760 | R_S_suborbital | 0.035810 |
| 107 | 0.05 | 0.149407 | R_G_Ins_IgS_cent_ins | 0.036149 |
| 108 | 0.05 | 0.153200 | L_S_octemp_lat | 0.036487 |
| 109 | 0.05 | 0.159817 | L_S_orbital_medolfact | 0.036824 |
| 110 | 0.05 | 0.165426 | R_G_postcentral | 0.037162 |
| 111 | 0.05 | 0.169522 | R_G_front_infTriangul | 0.037500 |
| 112 | 0.05 | 0.179232 | R_S_orbital_lateral | 0.037838 |
| 113 | 0.05 | 0.180962 | L_G_insular_short | 0.038176 |
| 114 | 0.05 | 0.196170 | L_Lat_FisantVertical | 0.038514 |
| 115 | 0.05 | 0.198483 | L_G_octemp_medParahip | 0.038851 |
| 116 | 0.05 | 0.224746 | R_S_occipital_ant | 0.039189 |
| 117 | 0.05 | 0.226275 | R_S_oc_middleLunatus | 0.039527 |
| 118 | 0.05 | 0.252851 | R_G_orbital | 0.039865 |
| 119 | 0.05 | 0.290544 | R_Lat_FisantHorizont | 0.040203 |
| 120 | 0.05 | 0.300996 | L_GS_cingulMidAnt | 0.040541 |
| 121 | 0.05 | 0.323948 | R_S_calcarine | 0.040878 |
| 122 | 0.05 | 0.339193 | L_G_rectus | 0.041216 |
| 123 | 0.05 | 0.346644 | L_S_pericallosal | 0.041554 |
| 124 | 0.05 | 0.366783 | L_Lat_FisantHorizont | 0.041892 |
| 125 | 0.05 | 0.367490 | R_S_circular_insula_inf | 0.042230 |
| 126 | 0.05 | 0.374609 | R_S_orbital_medolfact | 0.042568 |
| 127 | 0.05 | 0.407687 | R_GS_subcentral | 0.042905 |
| 128 | 0.05 | 0.409952 | L_S_central | 0.043243 |
| 129 | 0.05 | 0.431454 | R_S_circular_insula_ant | 0.043581 |
| 130 | 0.05 | 0.445615 | R_G_cingulPostventral | 0.043919 |
| 131 | 0.05 | 0.453596 | R_G_temp_supPlan_polar | 0.044257 |
| 132 | 0.05 | 0.478014 | L_S_calcarine | 0.044595 |
| 133 | 0.05 | 0.479667 | R_G_front_infOrbital | 0.044932 |
| 134 | 0.05 | 0.563455 | R_G_octemp_medParahip | 0.045270 |
| 135 | 0.05 | 0.595331 | L_S_collat_transv_ant | 0.045608 |
| 136 | 0.05 | 0.665290 | L_S_circular_insula_ant | 0.045946 |
| 137 | 0.05 | 0.727270 | R_S_interm_primJensen | 0.046284 |
| 138 | 0.05 | 0.734277 | L_G_temp_supPlan_polar | 0.046622 |
| 139 | 0.05 | 0.761508 | L_G_Ins_IgS_cent_ins | 0.046959 |
| 140 | 0.05 | 0.785965 | R_G_precentral | 0.047297 |
| 141 | 0.05 | 0.798275 | R_S_central | 0.047635 |
| 142 | 0.05 | 0.863660 | R_G_insular_short | 0.047973 |
| 143 | 0.05 | 0.873088 | R_Pole_temporal | 0.048310 |
| 144 | 0.05 | 0.896209 | R_S_pericallosal | 0.048649 |
| 145 | 0.05 | 0.913080 | L_G_subcallosal | 0.048987 |
| 146 | 0.05 | 0.951174 | L_Pole_temporal | 0.049324 |
| 147 | 0.05 | 0.955415 | R_S_collat_transv_ant | 0.049662 |
| 148 | 0.05 | 0.980884 | L_G_cingulPostventral | 0.050000 |
